# The Crystal Structure of *Thermotoga maritima* Class III Ribonucleotide Reductase Lacks a Radical Cysteine Pre-Positioned in the Active Site

**DOI:** 10.1371/journal.pone.0128199

**Published:** 2015-07-06

**Authors:** Oskar Aurelius, Renzo Johansson, Viktoria Bågenholm, Daniel Lundin, Fredrik Tholander, Alexander Balhuizen, Tobias Beck, Margareta Sahlin, Britt-Marie Sjöberg, Etienne Mulliez, Derek T. Logan

**Affiliations:** 1 Dept. of Biochemistry & Structural Biology, Lund University, Box 124, S-221 00 Lund, Sweden; 2 Dept. of Biochemistry & Biophysics, Stockholm University, S-106 91 Stockholm, Sweden; 3 Dept. of Medical Biochemistry and Biophysics, Karolinska Institute, Solna, Sweden; 4 Dept. of Inorganic Chemistry, Georg-August Universität Göttingen, Göttingen, Germany; 5 LCBM, Groupe de Biocatalyse, CEA-Grenoble, Institut de Recherches en Technologies et Sciences pour le Vivant (iRTSV), 38054 Grenoble Cedex 09, France; University of Nottingham, UNITED KINGDOM

## Abstract

Ribonucleotide reductases (RNRs) catalyze the reduction of ribonucleotides to deoxyribonucleotides, the building blocks for DNA synthesis, and are found in all but a few organisms. RNRs use radical chemistry to catalyze the reduction reaction. Despite RNR having evolved several mechanisms for generation of different kinds of essential radicals across a large evolutionary time frame, this initial radical is normally always channelled to a strictly conserved cysteine residue directly adjacent to the substrate for initiation of substrate reduction, and this cysteine has been found in the structures of all RNRs solved to date. We present the crystal structure of an anaerobic RNR from the extreme thermophile *Thermotoga maritima* (tmNrdD), alone and in several complexes, including with the allosteric effector dATP and its cognate substrate CTP. In the crystal structure of the enzyme as purified, tmNrdD lacks a cysteine for radical transfer to the substrate pre-positioned in the active site. Nevertheless activity assays using anaerobic cell extracts from *T*. *maritima* demonstrate that the class III RNR is enzymatically active. Other genetic and microbiological evidence is summarized indicating that the enzyme is important for *T*. *maritima*. Mutation of either of two cysteine residues in a disordered loop far from the active site results in inactive enzyme. We discuss the possible mechanisms for radical initiation of substrate reduction given the collected evidence from the crystal structure, our activity assays and other published work. Taken together, the results suggest either that initiation of substrate reduction may involve unprecedented conformational changes in the enzyme to bring one of these cysteine residues to the expected position, or that alternative routes for initiation of the RNR reduction reaction may exist. Finally, we present a phylogenetic analysis showing that the structure of tmNrdD is representative of a new RNR subclass IIIh, present in all Thermotoga species plus a wider group of bacteria from the distantly related phyla Firmicutes, Bacteroidetes and Proteobacteria.

## Introduction

**Fig 1:**
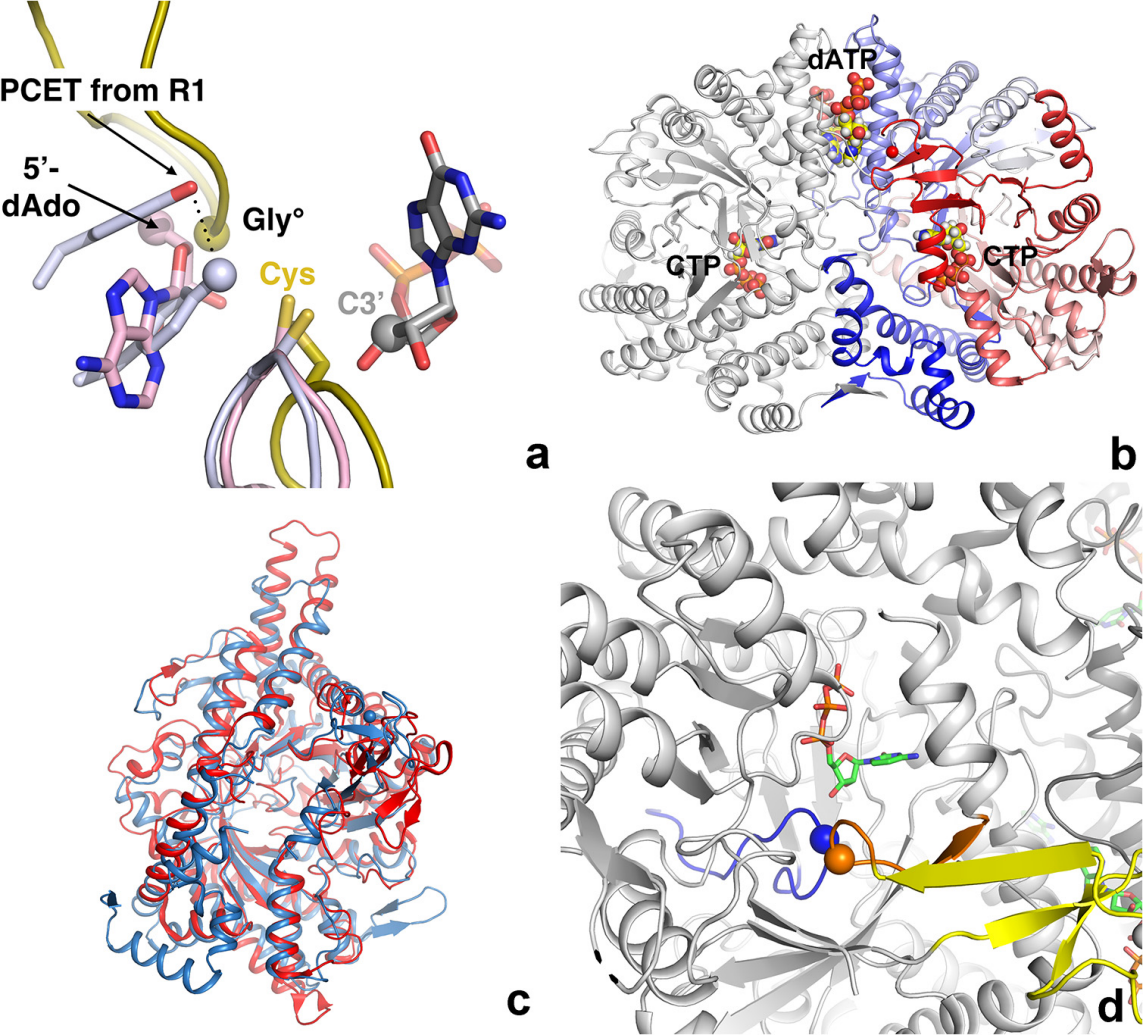
a) The radical generation and transfer pathways of all three classes of RNR are thought to converge on a completely conserved cysteine residue that transfers it to the substrate. The class I, II and III enzymes are coloured mauve, pink and gold respectively. The finger loops of all three classes and the C-terminal loop of the class III RNRs, as exemplified by the enzyme from bacteriophage T4, are shown in cartoon representation. The position of the glycyl radical in class III is marked by a sphere. The two hydrogen-bonded Tyr residues that end the proton-coupled electron transfer chain (PCET) in class I are shown in mauve, with the terminal oxygen atom shown as a sphere. The 5’-deoxyadenosine moiety generated by cleavage of the C-Co bond in AdoCbl by class II RNRs is shown with the 5’-C atom shown as a pink sphere. The GDP substrate bound to the class II enzyme is shown as sticks with the C3’ atom marked with a gray sphere. b) Overall structure of the tmNrdD dimer. The left-hand monomer is coloured grey while the right-hand monomer is coloured as a spectrum from deep blue at the N-terminus to deep red at the C-terminus. The allosteric effector dATP and the substrate CTP are shown in space-filling representation. c) Comparison of the structures of tmNrdD and the previously determined structure of NrdD from bacteriophage T4 [9]. The T4 structure is coloured dark blue and tmNrdD is coloured red. d) Depiction of the active site area where the tips of the finger loop (blue) and the C-terminal loop (orange) meet. The position of the glycyl radical is marked by an orange sphere and Ile359 at the tip of the finger loop by a blue sphere. The substrate CTP is shown in stick representation. The Zn-binding domain is shown in yellow.

Ribonucleotide reductases (RNRs) are highly important enzymes for all life, as they are solely responsible for the first committed step in the synthesis of the deoxyribonucleoside triphosphate (dNTP) building blocks of DNA [1]. Using three different types of radical chemistry they catalyze the reduction of either nucleoside diphosphates (NDP to dNDP) or nucleoside triphosphates (NTPs to dNTPs) [1]. RNRs are commonly divided into three main classes based on this radical chemistry, as well as their 3D structures, expression conditions and cofactor requirements: class I RNRs are dependent on a dinuclear metal cofactor and molecular O_2_ in almost all cases for generation of a stable tyrosyl radical [2]. The radical is then transferred to the active site through a 30–35 Å long proton-coupled electron transfer pathway terminating in a pair of Tyr residues in the active site of the reductase (Fig 1A). Class II RNRs, indifferent to oxygen levels, generate a 5’-deoxyadenosyl (5’-dAdo) radical through homolytic cleavage of the C-Co bond in their adenosylcobalamin cofactor [3,4]. Class III RNRs, strictly anaerobic, generate on a separate activating enzyme NrdG the same type of 5’-dAdo radical through homolytic cleavage of a C-S bond in S-adenosylmethionine, with the participation of a [4Fe-4S] cluster [5,6]. The 5’-dAdo radical in turn abstracts an H atom from a glycine residue in a C-terminal, inward-pointing loop in the reductase NrdD [7–9]. This glycyl radical is stable and can catalyze several dozen cycles before having to be regenerated [10,11].More than five decades of research on RNRs, including the crystal structure determination of several RNRs representing all three classes [9,12–14], have led to the insight that, despite often significant differences in sequence and radical generation mechanism, all RNRs are characterized by a 10-stranded α-β barrel fold containing at its heart the “finger loop” [12]. This loop harbors a critical, highly conserved cysteine residue responsible for initiation of the reduction reaction by abstraction of a hydrogen atom from the 3’ carbon of the substrate ribose ring [15]. The RNR fold is shared by other enzymes employing glycyl radicals, such as pyruvate formate lyase (PFL), glycerol dehydratase (GD) [16,17], 4-hydroxyphenylacetate decarboxylase (4-HPAD) [18] and benzylsuccinate synthase [19]. Two other active site cysteines on neighboring β-strands, conserved in class I and II RNRs, are responsible for electron and proton donation to the substrate during the reduction reaction and in the process form a disulphide bond that is indirectly reduced by thioredoxin (Trx) [20,21]. Only one of the latter two cysteines is conserved in class III RNR [9] and both reducing equivalents are provided by the small cosubstrate formate [22] in most systems characterized to date, although very recently Trx was also shown to be a possible reductant for the NrdD from *Neisseria bacilliformis* [23]. Nevertheless, whatever the means by which the stable or transient radicals described above are generated, it is thought that the single unifying factor of RNR radical chemistry is the transfer of these radicals to the critical cysteine at the initiation of catalysis [24] (Fig 1A). This cysteine is thought to be completely conserved across all RNRs, having been found in the sequences and structures of RNRs from all classes.

The first crystal structure of an class III RNR to be determined was that of the enzyme from bacteriophage T4 (T4NrdD [9]). This structure contains the expected critical cysteine. Here we present the crystal structure of a second class III RNR from *Thermotoga maritima* (tmNrdD), which, as purified and crystallized, surprisingly does not display such a cysteine, possessing an isoleucine (Ile) in its place. The sequence where the initiator cysteine was expected is found in a disordered loop on the far side of the protein. Nevertheless tmNrdD binds a substrate and its allosteric effector in productive conformations as expected from previous structures of RNRs of all three classes, and it is enzymatically active. The sequence characteristics of tmNrdD, including a hydrophobic residue in the same position as Ile in tmNrdD, are found in a significant number of other class III RNRs, which we propose form a new RNR subclass: IIIh (see RNRdb2, currently in beta at http://rnrdb.pfitmap.org). This strongly suggests that the features seen in tmNrdD may be more general and that our view of the initiation of substrate reduction in RNRs may have to be widened to include the possibility that a radical initiator cysteine is introduced to the active site by large conformational changes, or alternatively that a novel activation mechanism is operative.

## Materials and Methods

Native and SeMet-derivatized proteins were produced by heterologous overexpression in *E*. *coli* and purified by hydrophobic interaction and gel filtration chromatography. Initial crystallization conditions were found using commercial screens in 96-well plates and optimized in a variety of different crystallization setups. SeMet multiple wavelength anomalous diffraction (MAD) and native/citrate/PEG400 data were collected at stations ID23-1 and ID23-2 of the European Synchrotron Radiation Facility (ESRF) in Grenoble, France [25] respectively. All other diffraction data were collected at stations I911-2 and I911-3 of the MAX IV Laboratory, Lund, Sweden [26]. The structure was solved by the MAD method using three wavelengths and the AutoRickshaw pipeline [27]. Structures of nucleotide complexes were obtained by co-crystallization or soaking with 0.5 mM dATP and 2 mM CTP. Full methods, including production and reconstitution of active MBP-tmNrdG, EPR spectra, activity assays and bioinformatics analyses, can be found in S1 Text, S1 Table and S2 Table. The following structures have been deposited in the Protein Data Bank: tmNrdD in complex with glycerol (4COI), MES (4COM), citrate (4CON), dATP/CTP (4COJ) and dATP only (4COL).

## Results

### Overall structure of tmNrdD

The structure of the anaerobic ribonucleotide reductase from *Thermotoga maritima* (tmNrdD; Uniprot ID Q9WYL6) was solved by the MAD method from a SeMet derivative, in its apo form and in various complexes, including with the effector dATP and the effector/substrate combination dATP/CTP, at resolutions from 2.4 Å to 1.92 Å. The crystallographic and geometric quality indicators are good (S2 Table). The crystals contain a tmNrdD dimer in the asymmetric unit (Fig 1B) and the structure is essentially identical in both monomers in all structures. The native structure was solved from crystals obtained under three different conditions, one with the buffer molecule citrate bound in the active site, another with MES and a third with glycerol (S1 Fig). It was possible to model 95% of the structure of both chains in the electron density, with only three disordered regions: loop 50–61, loop 331–349 and the C-terminal 17 residues (634–651). TmNrdD possesses the 10-stranded α-β barrel fold with a “finger loop” wound through its core, a fold common to RNR and other glycyl radical enzymes [28]. The root mean square deviation (rmsd) to the previously determined crystal structure of the bacteriophage T4 enzyme [T4NrdD, PDB ID 1HK8] [29] is 2.34 Å for 426 equivalent Cα atoms out of 505 in T4NrdD and 606 in tmNrdD (Fig 1C). This large structural deviation is consistent with the very limited sequence identity of ~18% between the two proteins. A full comparison of all known structures of RNR large subunits is given in S3 Table. The tmNrdD structure was also very recently solved by another group [23] (PDB ID 4U3E). The crystal form these workers obtained was very similar, but due to subtly different crystal packing they were able to resolve a further 17 residues at the C-terminus of chain A and 4 in chain B. However the C-termini of chain A packs against the same region of chain B in a symmetry-related molecule in the crystal, thus the conformations of both C-termini in 4U3E are determined by crystal packing and their biological relevance is not clear.

As in T4NrdD [30], the α-β barrel is decorated by an extra Zn-ribbon domain inserted between the last strand of the barrel and the C-terminal loop containing the glycyl radical site (S2 Fig). The identity of the metal ion has been confirmed by anomalous diffraction at the Zn and Fe K edges (S1 Table and S2 Fig). The Zn^2+^ ion is coordinated by the motif CxxxHx_6_CxxC [CxxCx_14_CxxC in the T4 and *E*. *coli* enzymes]. This motif is representative of 87 of the 1502 NrdD sequences in the RNR database RNRdb [31] (RNRdb2 beta, http://rnrdb.pfitmap.org; only NCBI sequences from organisms with fully sequenced genomes counted). The Zn-binding domain is more compact than in T4NrdD due to the shorter loop joining the Zn ligands. It is also oriented differently with respect to the barrel surface (S2 Fig). On the basis of our earlier conjecture that this domain is involved in interactions with the activase NrdG [30], we assume that these differences are due to different sizes and structures of NrdG in the two organisms (183 amino acids in tmNrdG vs. 156 in T4NrdG).

**Fig 2:**
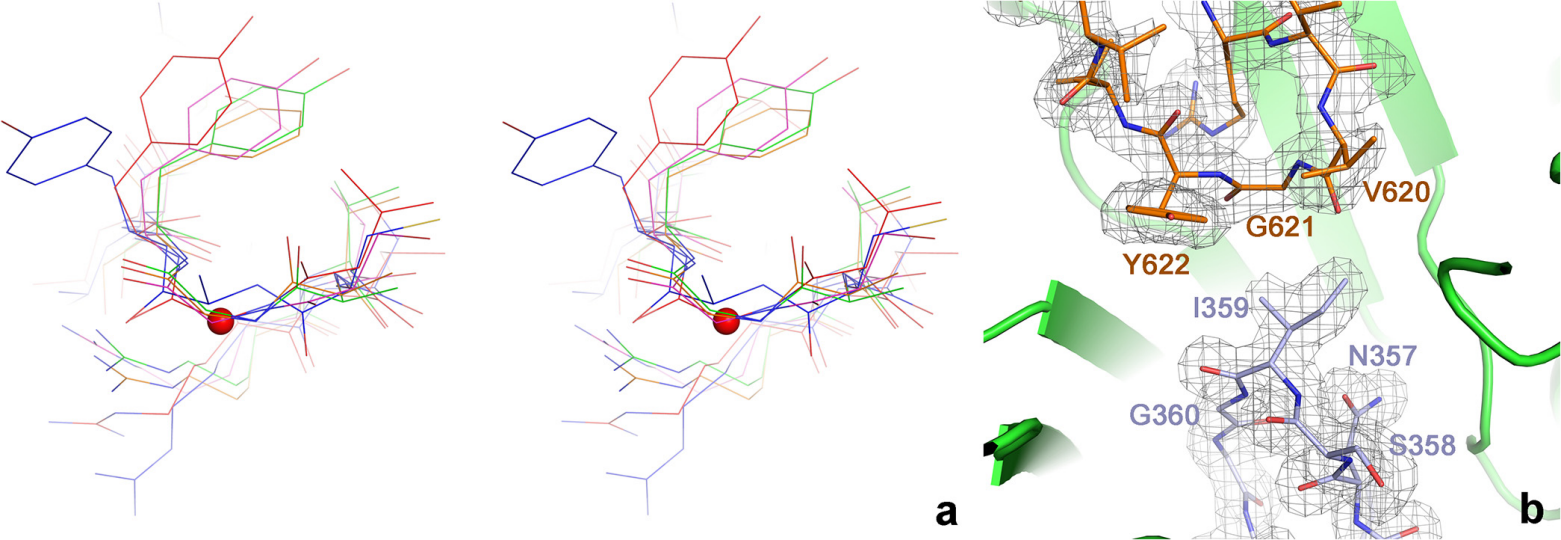
a) Details of the conformation of the glycyl radical and finger loops in the active site of tmNrdD. The two loops are shown as sticks with the surrounding **β**-barrel as a cartoon. A 2m|Fo|-D|Fc| omit electron density map is shown contoured at 1.0 σ. The map was calculated by omitting residues 351–364 and 618–623 from the model followed by three macrocycles of torsion angle molecular dynamics in phenix.refine with default parameters. b) Cross-eyed stereo view comparing the glycyl radical loops in several representative glycyl radical enzymes. tmNrdD is shown in red, T4NrdD in blue, pyruvate formate lyase in magenta, glycerol dehydratase in green and 4-hydroxyphenylacetate decarboxylase in orange. The C**α** atom of Gly621 in tmNrdD is indicated with a red sphere.

The tmNrdD structure contains a C-terminal loop harbouring the glycyl radical site (Gly621), which dips into the α-β barrel such that its tip meets that of the finger loop in the active site (Fig 1D). The electron density for both loops is very well defined (Fig 2A). Since the T4 enzyme structure was solved as an oxygen-insensitive Gly->Ala mutant [9], the present structure allows the native conformation of the radical Gly in an anaerobic ribonucleotide reductase to be observed in its resting state prior to activation, as also seen by Wei et al. [23]. The flanking sequence and conformation around Gly621 are very similar to those in the G580A mutant of T4NrdD, as well as PFL [32], GD [17] and 4-HPAD [18] (Fig 2B). Gly621 itself is completely buried from solvent and presumably undergoes a significant conformational change in order for its hydrogen atom to be abstracted by the 5’-dAdo radical generated by the activase, as postulated previously for T4NrdD [9] and PFL [33]. This is almost certainly linked to conformational changes in the entire C-terminal domain, which includes the Zn-binding domain, but comparison with the T4NrdD structure does not allow us to speculate whether the different orientations of the Zn domain are due to an inherent flexibility or entirely due to species variation.

### Lack of a radical cysteine at the tip of the finger loop

Remarkably, the residue at the tip of the finger loop in the crystal structure of tmNrdD is not, as expected, a cysteine, but rather an isoleucine (Ile359). The presence of this apparently inert residue at a key position for the RNR chemistry is highly unexpected; however the electron density allows unambiguous placement of an Ile here (Fig 2A). The side chain is positioned such that two of its atoms, C_δ1_ and C_**γ**2_, are in van der Waals contact with the C_α_ atom of Gly621, at distances of 3.3 Å and 3.8 Å respectively. The finger loop is held in place by hydrophobic and polar interactions with the inner sides of the β barrel and the Gly radical loop (S7A Fig), although many of the interactions are made through water molecules and few are sequence-specific. The number of water molecules inside the barrel is significantly higher than in T4NrdD (S7B Fig). Ile359 is completely buried and is not more mobile than the protein as a whole, having atomic B-factors under average for the structure as a whole (26 Å^2^ average in chains A/B vs. 31 Å^2^ for all protein main chain atoms in the native/MES structure; the corresponding values for the dATP/CTP complex are 41 Å^2^ and 50 Å^2^ respectively). Interestingly, although the finger loop in tmNrdD is structurally conserved with respect to T4NrdD, it ascends and descends the (α/β)_10_ barrel on opposite sides from T4NrdD, any other RNR or glycyl radical enzyme, which has the effect that the sequence order of residues at the tip of the loop as it passes the substrate is reversed compared to the other enzymes (S3 Fig).

**Fig 3:**
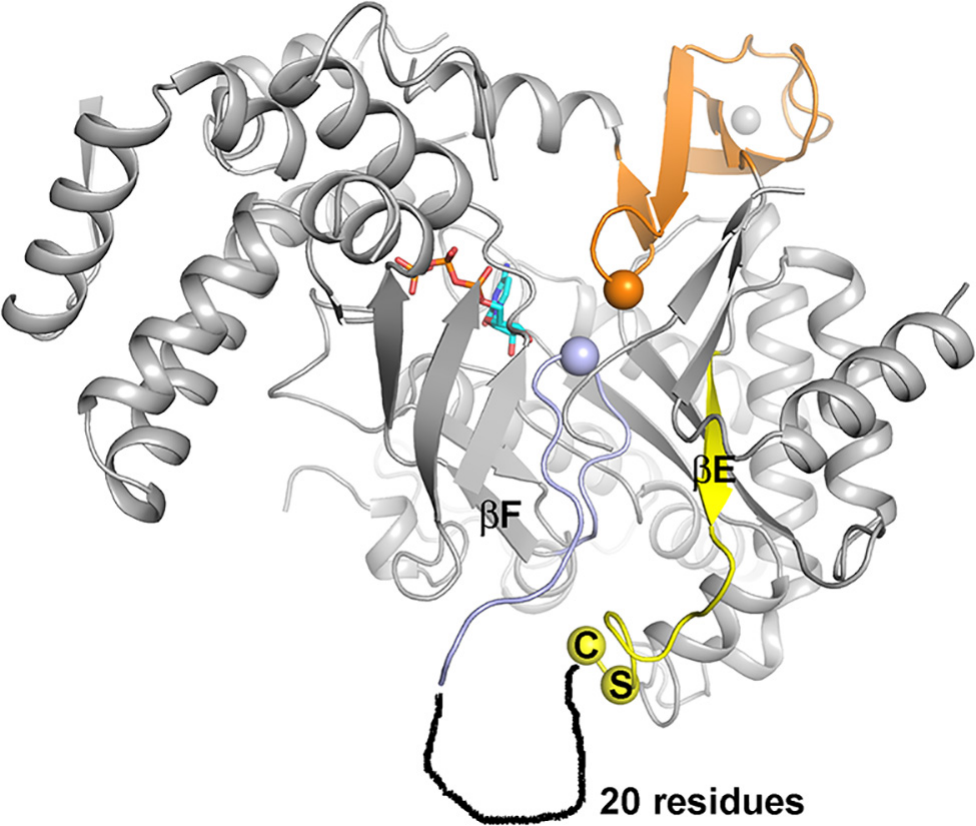
Position of the SCCR motif in relation to the active site in tmNrdD. The C-terminal domain and glycyl radical loop are shown in orange and the finger loop in light blue, with the Gly and Ile residues at their respective tips shown as spheres. The two residues Ser and Cys at the beginning of the SCCR motif are also pinpointed by spheres. The preceding **β**-strand **β**E is drawn in yellow and the approximate location of a disordered 20-residue segment following the SCCR motif is sketched. For clarity several **α**-helices on the front of the barrel have been removed.

The sequence that multiple sequence alignment programs typically align with the tip of the finger loop in the absence of structural guidance, ^328^SCCR^331^ (^288^MGCR^291^ in T4) is found in an extended loop region on the far side of the α-β barrel from the active site (Fig 3). The structure becomes disordered immediately after the first cysteine in the SCCR motif, Cys329, and the rest of the loop has the sequence characteristics of an intrinsically disordered region.

### Substrate and effector binding to a class III RNR

**Fig 4:**
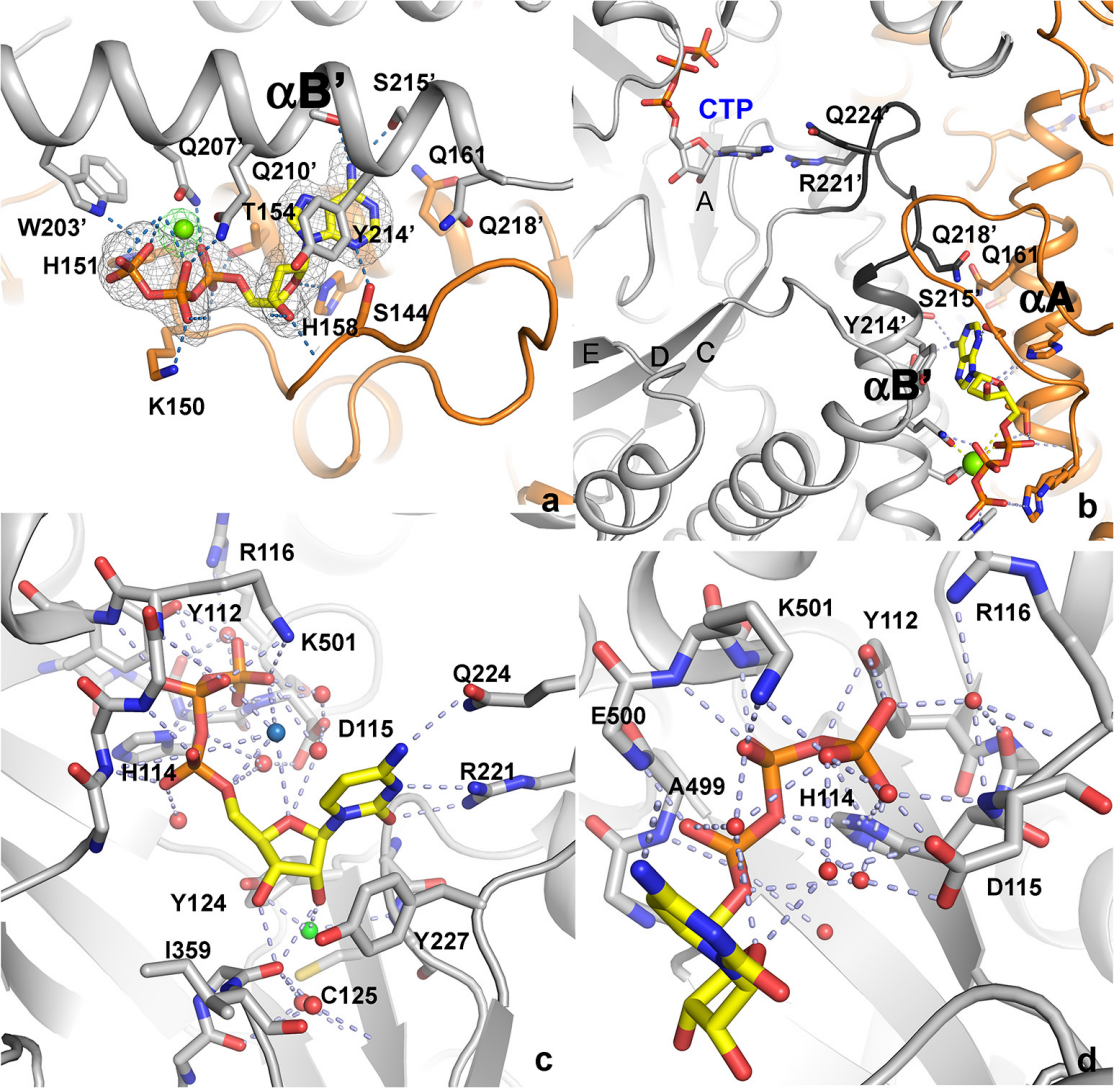
a) Details of the interactions of the allosteric effector dATP. The Mg^2+^ ion is shown as a green sphere. Monomer A of tmNrdD is coloured orange and monomer B is coloured grey. Hydrogen bonds to the protein are shown as dotted lines. An anomalous difference map generated by substitution of the Mg^2+^ ion by Mn^2+^ and collection of data near the Mn^2+^ K-edge (1.86 Å) is shown as green chicken wire. The contour level is 5.0 σ. An m|F_o_|-D|F_c_| omit map generated by excluding the coordinates of dATP followed by torsion angle simulated annealing in phenix.refine is shown as grey mesh, contoured at 3.0 σ. b) Communication pathway between allosteric effector dATP and substrate CTP. Both are shown in stick representation, as are the critical side chains that read out the effector base and those that make H-bonds to the substrate. Colouring is as in panel a) but loop 2 is coloured a darker shade of gray. c) Overall view of the interactions of CTP with tmNrdD. Hydrogen bonds are shown as dotted lines and water molecules involved in the network as small red spheres. The water molecule that lies between the CTP 2’-OH group and Cys125 is coloured green and the one in the bend of the triphosphate moiety discussed in the text is coloured pale blue. d) Close-up of the interactions of the CTP phosphate moieties with tmNrdD. Important hydrogen bonds are shown as dark dotted lines and water molecules involved in the interactions as small red spheres.

The structure of tmNrdD has also been determined in complex with the allosteric substrate specificity effector dATP and its cognate substrate CTP (Fig 4), as well as with dATP alone. The effector dATP binds at the dimer interface, along the length of helices αA and αB of the α-β barrel (Figs 1B, 4A & 4B; the naming of secondary structure elements follows the convention for T4NrdD [9]). The dATP base points into a pocket containing two glutamine residues: Gln161 from one chain and Gln218 from the other. Of these Gln161 is conserved in T4NrdD and Gln218 is conservatively substituted to Glu. In T4NrdD these residues change conformation in detailed response to the H-bonding patterns presented by the effector base, inducing conformational changes in loop 2 that lead to altered specificity [29]. The conservation of Gln residues for effector recognition in evolutionarily distantly related RNRs, with their ability of the side chain to present H-bond donors or acceptors to the effector base by simple flipping, is interesting; however in tmNrdD neither of these critical residues H-bonds directly to the dATP base, suggesting that the mechanisms of allosteric specificity regulation in these distantly related class III RNRs are subtly different. A full analysis of the mechanism of allosteric specificity regulation will be presented elsewhere. Specificity effector binding involves a Mg^2+^ ion [29]. For T4NrdD the electron density was ambiguous and allowed the building of the effector phosphate groups in two possible conformations. In the present work we have identified the dNTP conformation unambiguously for tmNrdD by substituting Mn^2+^ for Mg^2+^ and collecting anomalous diffraction data (Fig 4A) at the Mn K edge. This places the metal ion in proximity to Gln210 and thus corrects the interpretation we made previously for T4NrdD [29], where the β-phosphate group was placed in this position. It cannot be excluded that the difference is species-specific, but we consider this possibility unlikely.

The dATP/CTP complex represents the first structure of an anaerobic, class III RNR with a substrate and the first of any RNR in complex with a triphosphate substrate. The electron density is very well defined for all parts of the CTP nucleotide (S4 Fig). The average B-factor of CTP in chain A is 39 Å^2^ and in chain B it is 52 Å^2^, which is comparable to the average 49 Å^2^ for all protein atoms (identical for chains A and B). The cytosine base stacks imperfectly on top of Tyr227, equivalent to Phe194 in T4NrdD. This orients the base towards the flexible loop 2 (residues 217–228), which has been observed to change conformation in all three RNR classes in response to specificity effector binding [14,29,34–36]. The shortest distance between the bases of effector and substrate is about 21 Å. Effector binding induces a conformation of the substrate-proximal side of loop 2 that projects the side chains of Arg221 and Gln224 towards the substrate base (Fig 4B and 4C), thereby enhancing CTP binding through three H-bonds between these side chains and its base (Fig 4C). Full details of effector and substrate identity recognition will be presented elsewhere.

The dATP/CTP complex confirms that the binding mode of effectors and substrates to class III RNRs is very similar to that seen in classes I and II [14,21,35–37], allowing a comparison of all RNR classes. As in the other classes, the α and β phosphate groups of the substrate are held in place by several H-bonding interactions to the N-terminus of a conserved α-helix (499–514; Fig 4C and 4D). Three of the four potential negative charges on CTP are compensated for by the dipole of helix 499–514, Arg116, Lys501 and His114 (assuming that the latter is protonated). The γ-phosphate group interacts with the side chain hydroxyl group of Tyr112, His114 and the main chain amide group of Asp115. Tyr112 and His114 are highly conserved in NrdD sequences, although Tyr112 is more often His. Thus the consensus sequence motif for triphosphate rather than diphosphate binding to class III RNRs can be defined as ^111^HYHD^115^ (tmNrdD numbering). The highly conserved Asp115 coordinates a water molecule that binds in the “bend” of the three phosphate groups and stabilizes the CTP conformation (pale blue sphere in Fig 4C). It seems unlikely that this water molecule is a Mg^2+^ ion, as is the case with the effector dATP, as the coordination distances to oxygen atoms in the CTP phosphate groups are too long, at around 3 Å, and coordination distances for Mg^2+^ should be around 2.0–2.1 Å. We performed test refinements where each of the water molecules near CTP in both monomers were replaced by Mg^2+^ ions, and in all cases the B-factors increased, by 16–44%, and the atomic positions did not adjust to be more compatible with Mg^2+^ coordination by oxygen atoms on CTP.

The substrate ribose is oriented by an H-bond between O3’ and the main chain carbonyl atom of Ser358 in the finger loop, as well as between O2’ and the main chain amide nitrogen of Ala173 in strand βB, through a water molecule (green sphere in Fig 4C)**.** These interactions replace those between the substrate and a conserved glutamate and asparagine in class I and II RNR (Glu441 and Asn437 in *E*. *coli* class I RNR [21]). Cys125, which is completely conserved in all known RNRs and is one of the two cysteines oxidized by an intermediate substrate radical to form a disulphide radical during the RNR reaction in class I/II, has its sulphur atom around 6 Å from O2’ and is separated from it by a water molecule. This implies either that electron transfer from Cys125 to the substrate is indirect or that Cys125 may change conformation during the reaction cycle. Interestingly, the side chain of Tyr124, completely conserved in subclass NrdDh (see below) is oriented such that its terminal hydroxyl atom is poised approximately equidistant from the O2’ and O3’ atoms of the ribose (Fig 4C). Tyr124 is equivalent to residue Asn78 in T4NrdD, mutation of which leads to loss of activity [38]. It was suggested that these residues might form part of a binding site for the cofactor formate in T4NrdD [38]; however for steric reasons this role seems unlikely for Tyr124 in tmNrdD. Most likely formate does not have a fixed binding site in the active sites of class III RNRs.

### The class III RNR from *T*. *maritima* is enzymatically active

**Fig 5:**
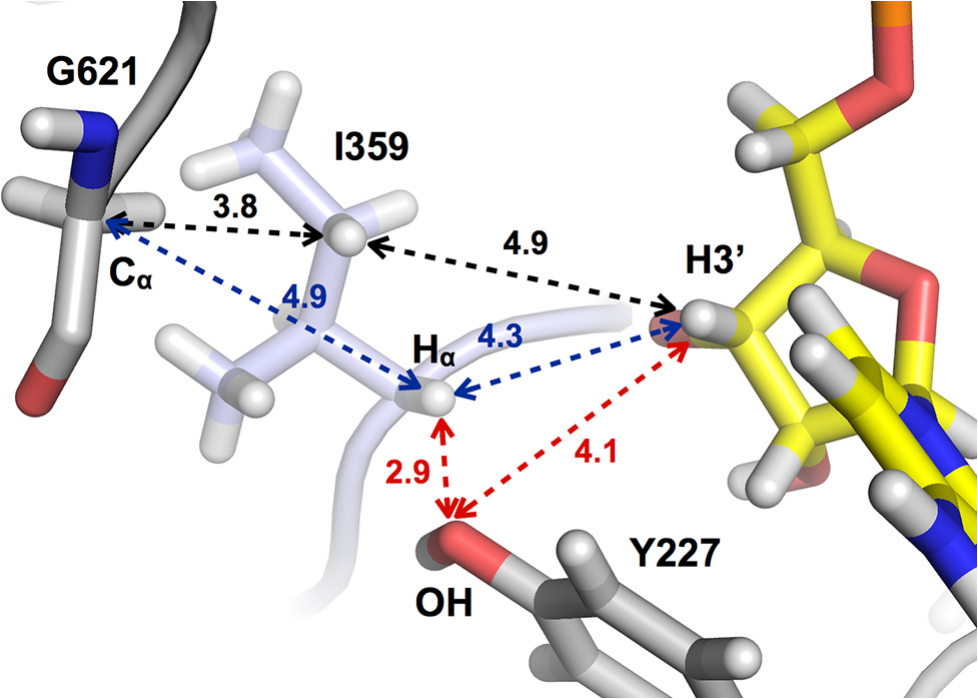
Schematic of key distances between the Cα atom of Gly621, atoms in Ile359 and the H3’ atom of the substrate CTP. Distances between key atoms are shown in Å.

**Fig 6:**
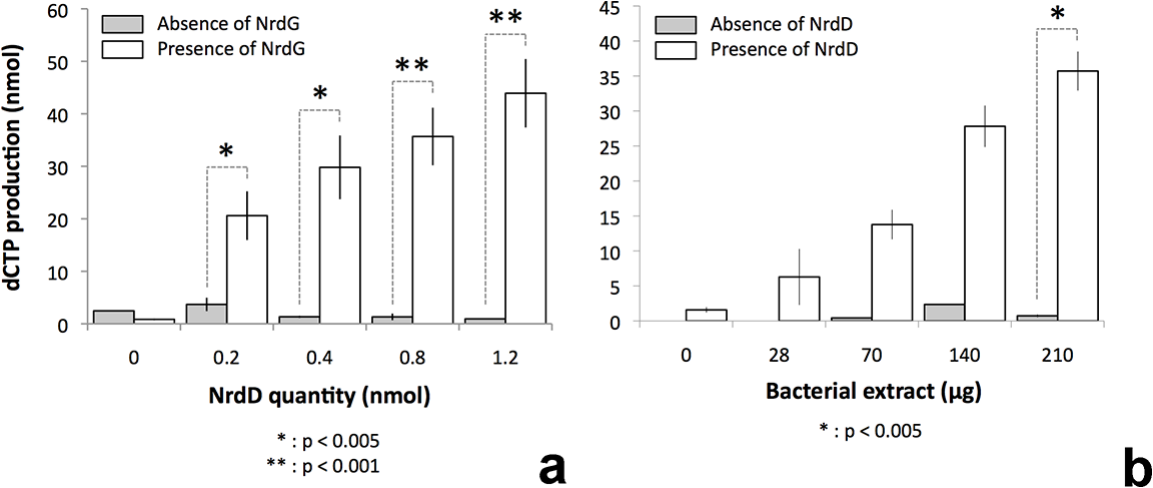
Activity assays for the *T*. *maritima* anaerobic RNR carried out in the presence of anaerobically prepared cell extracts. a) White bars show activity in the presence of fixed amounts of cell extract and MBP-tagged tmNrdG and increasing amounts of tmNrdD. Grey bars show the corresponding activity in the absence of tmNrdG. b) activity in the presence of fixed amounts of tmNrdG and tmNrdD (0.4 nmol) and increasing amounts of *T*. *maritima* cell extract. White bars show the activity in the presence of tmNrdD and grey bars the corresponding activity in the absence of tmNrdD. The results are from 3–5 experiments, some performed with different protein preparations. All values are given as the mean +/- standard deviation. Statistical analysis was performed using the standard Student T-test.

With the ribose positioned as described, Ile359 at the tip of the finger loop is positioned in between the radical-bearing residue Gly621 and the substrate (Fig 5); however it does not reveal an obvious pathway for transfer of the radical (see Discussion). The absence of Cys at a critical position in the radical transfer pathway raised the question whether tmNrdD was a functional RNR. Initially, activity assays were hampered by the fact that the native activase tmNrdG displayed very limited solubility at pH 7.0, which is required for reconstitution of its [4Fe-4S] center. In order to overcome this we generated tmNrdG tagged with maltose binding protein (MBP) and using this system in combination with photoactivation using deazaflavin we were able to generate a glycyl radical on tmNrdD (S5 Fig). This radical has a g-value of 2.0056, which is comparable to that of Gly radicals in other anaerobic RNRs from bacteriophage T4 (2.0039) [8], *E*. *coli* (2.0033) [7,39], *Lactococcus lactis* (2.0033) [40] and *N*. *bacilliformis* (2.0076) [23]. The glycyl radical content, estimated using a Cu^2+^ standard, is about 0.15 Gly° per monomer. We do not observe the fine structure seen in the EPR spectrum of the radical from *N*. *bacilliformis* [23], similar to that seen in PFL [41] and attributed to hyperfine coupling to the α-protons of adjacent residues. Presumably the difference between *T*. *maritima* and *N*. *bacilliformis* is due to differences in the local conformation of the glycyl radical loop. Generation of the glycyl radical in D_2_O resulted in no change in the shape of the spectrum, consistent with the fact that the hydrogen atoms of the radical Gly in NrdD are not expected to exchange with solvent [39]. Subsequent to generation of the glycyl radical we carried out anaerobic activity assays in order to probe whether the Gly radical could be transferred to the substrate. Initial trials using formate as reductant and ^3^H-labeled CTP as substrate were unsuccessful. However activity was obtained using anaerobically prepared extracts of *T*. *maritima* (Fig 6). These assays showed increasing triphosphate reductase activity as a function of the concentration of tmNrdD or cell extracts in the presence of fixed amounts of the other components. Control experiments omitting either tmNrdD or tmNrdG from the reaction mixture showed only negligible activity (Fig 6). Both assays tail off at the highest concentrations of the titrated component, most likely because of exhaustion of one of the non-protein assay components. The turnover number can be calculated from the data in Fig 6 and is around 0.4 s^-1.^ Thus tmNrdD is about 5–10 times less active than the *E*. *coli* [11], *Lactococcus lactis* [10] and bacteriophage T4 [8] enzymes and twice as active as that of *N*. *bacilliformis* [23]. However it should be borne in mind that the assays have been done with *T*. *maritima* cell extracts and not with purified components, and that they have been done at 37°C, while the optimal growth temperature of *T*. *maritima* is 80°C [42].

When assays were carried out using cell extracts passed through a filter with 10 kDa cutoff, the critical component was found to reside in the high molecular weight fraction, indicating that it is a protein and not a small molecule. The predicted molecular weight of *T*. *maritima* thioredoxin is 24.1 kDa. We tested the ability of the *E*. *coli* Trx system (MW 11.8 kDa) to substitute for anaerobic *T*. *maritima* cell extracts, as the *N*. *bacilliformis* NrdD could use *E*. *coli* Trx as reductant [23], but we found that it was unable to do so. Most likely this is due to very different sizes and limited sequence identity (21%) between *E*. *coli* and *T*. *maritima* thioredoxins.

### Mutation of the cysteines in the SCCR motif results in inactive enzymes

In order to investigate why the SCCR motif, upstream of the finger loop as observed in the tmNrdD structure, was highly conserved despite its location remote from the active site, and whether it may be involved in catalysis, we generated the mutants C329A, C330A and the double mutant C329A/C330A. Activity assays using anaerobic cell extracts showed that C329A and C330A have activities virtually indistinguishable from the background detected without addition of tmNrdD to the reaction mixture (Table 1). These results indicate, intriguingly, that these residues are critical for CTP reduction activity, although they are found far from the active site in the crystal structure.

**Table 1:** Activity assays on mutants of cysteine residues in the SCCR motif.

	Experiment 1	Experiment 2	
	^3^H-CTP assay [nmol*mg^-1^*min^-1^]	^3^H-CTP assay [nmol*mg^-1^*min^-1^]	Cold CTP assay [nmol*mg^-1^*min^-1^] (standard deviation)
Wild type	9.6 (± 1.1) [2]	8.4	7.2 (± 0.5) [3]
C329A	0.0	0.1	ND [3]
C330A	0.3	0.1	ND [3]
C329A / C330A	0.1	0.1	ND [3]

Amount of dCTP in nmol formed per minute and milligram of tmNrdD in the cysteine mutants compared to wild type tmNrdD. The experiments were done in two separate laboratories under different conditions. Conditions for experiment 1/2: 20/40 minute assay at 37/27°C with 20/5 μM tmNrdD and 2.5/5 μM MBP-tmNrdG in 100/80 μl reactions. Detailed assay conditions are listed in SI. ND = no dCTP detected. The detection limit of the HPLC assay is 10 pmol dCTP and the linear range is 10–500 pmol. For the ^3^H-CTP assay the detection limit is approx. 35 pmol. Numbers in square brackets indicate the number of replicates, where >1. For the ^3^H-CTP assay the activity measured for the mutants was too low to calculate an error.

### tmNrdD exemplifies a new subclass of anaerobic RNRs

**Fig 7:**
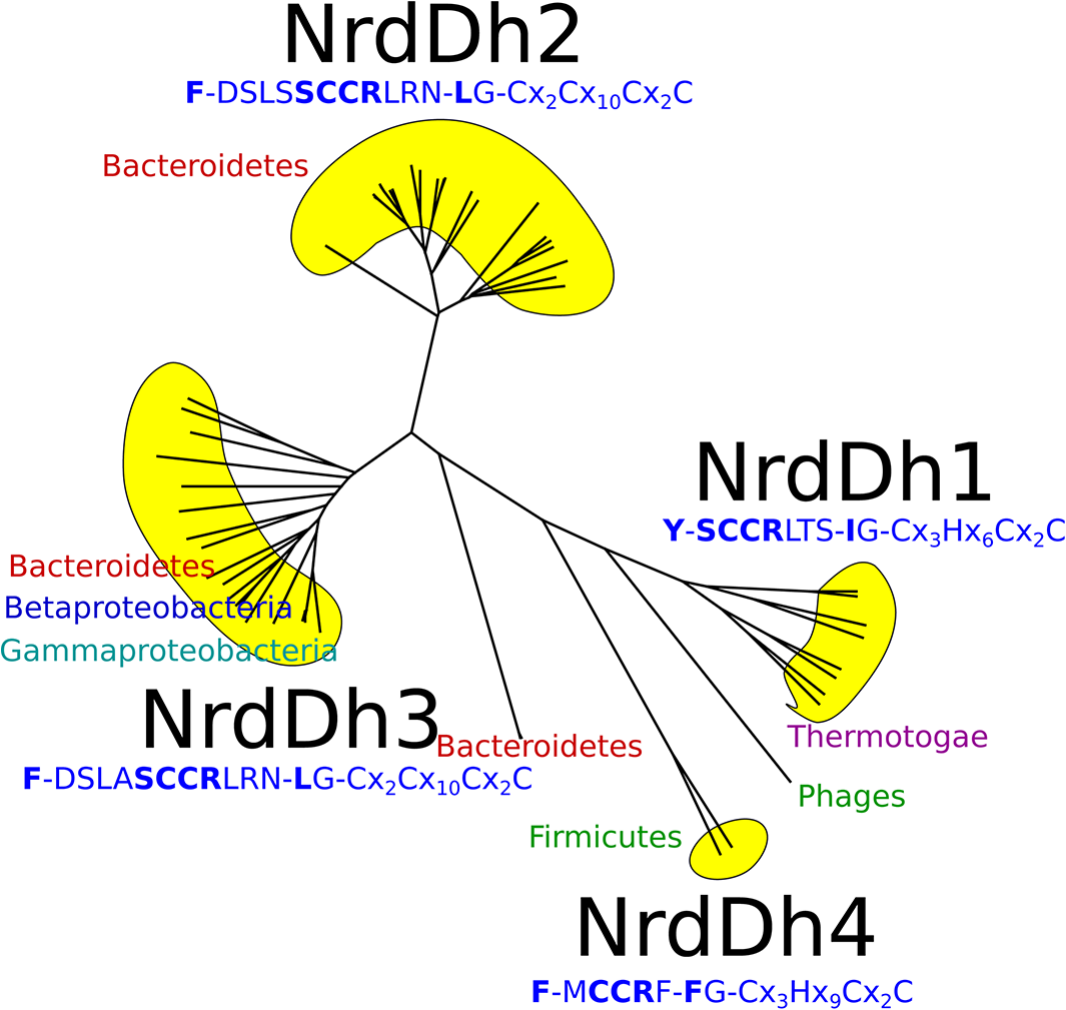
Unlabelled phylogenetic tree for the NrdDh class of NrdD sequences. The characteristic sequence motifs for each of the subgroups NrdDh1-4 are shown, along with the phyla. For more details, including the presence of other RNR classes in the genomes of the organisms, see S6 Fig.

To understand how widespread this class of RNR might be, we performed sequence analysis using hidden Markov models from RNRdb2 (http://rnrdb.pfitmap.org). In RNRdb2, the highly diverse RNR protein family is divided into subclasses based on phylogenetic evidence [43]. Subclass NrdDh, to which tmNrdD belongs, possesses a hydrophobic residue equivalent to Ile359. The organization of NrdDh is exemplified by the tmNrdD structure, and the subclass can be further subdivided into four groups based on sequence similarity: NrdDh1-4 (Fig 7 and S6 Fig). For comparison, the T4NrdD structure belongs to subclass IIIc (see RNRdb2). NrdDh is encoded and evolutionarily conserved in genomes with a phylogenetically wide distribution–two proteobacterial classes, Firmicutes and Bacteroidetes in addition to Thermotogae. The Ile at position 359 in tmNrdD is shared by all NrdDh1 sequences, all from Thermotogales except two from Clostridium phages, but is Leu in NrdDh2 and -3, and Phe in NrdDh4 (Fig 7 and S6 Fig), although an insert prior to the Ile in the NrdDh1 sequences creates some uncertainty as to exactly which residue is homologous to the tmNrdDh Ile in the other three groups. Another characteristic of the NrdDh1 group is a Tyr at position 227, which replaces the Phe conserved in other NrdDh as well as all other NrdDs. All other NrdDh sequences identified by HMMER using specific profiles for NrdDh possess the SCCR motif–except NrdDh4 which has a Met instead of Ser–in combination with the putative finger loop motif containing either Leu or Phe instead of Ile (Fig 7 and S6 Fig). We have not found this motif outside of NrdDh. The SCCR motif is sandwiched in between other well defined, conserved sequence motifs defining strands βE and βF, and we predict that the SCCR sequence may be on the far side of the barrel in these structures as well.

## Discussion

The reaction mechanism first proposed for RNRs in the early 1980s has, with only minor modifications, withstood three decades of experimental investigation [15]. It is based on the paradigm that initiation of ribonucleotide reduction is triggered by a transient Cys radical positioned at the tip of the finger loop, which abstracts an H-atom from the 3’ carbon atom of the substrate, irrespective of the way in which this radical was generated. A Cys residue with potential to bear such a radical has been present in the crystal structure of every RNR determined to date, and it was also assumed to be present in the active site of tmNrdD, as multiple sequence alignments identified it in the conserved sequence ^328^SCCR^331^ (tmNrdD numbering). However in the crystal structure of tmNrdD, determined independently by us and by another group [23], the SCCR motif is found on the far side of the tmNrdD barrel (Fig 2) in the enzyme as purified and crystallized, in a poorly ordered loop. Furthermore the expected radical Cys is replaced by a sequence containing Ile in direct proximity to the substrate. Nevertheless, the ability to generate a Gly radical in tmNrdD and enzymatic assays in the presence of anaerobically prepared cell extracts of *T*. *maritima* show that tmNrdD is active in spite of the absence of a Cys in the critical position in the crystal structure. Furthermore much evidence indicates that tmNrdD is functionally important for *T*. *maritima*. Firstly, peptides corresponding to tmNrdD can be found in mass spectrometry analyses of extracts of *T*. *maritima* from log- and mid-exponential phase, and their abundance is above the average for all expressed proteins [see Table S9 in [44]]. Secondly, studies of gene expression levels in *T*. *maritima* during hypothermic coculture with *Methanocaldococcus jannaschii* [45] or after chloramphenicol challenge [46] reveal significant drops in the levels of tmNrdD and tmNrdG expression compared to the levels in pure untreated mid-log cultures. Thirdly, tmNrdD, tmNrdG and the class II RNR tmNrdJ are all predicted by the RegPrecise database [47] to fall under the control of the transcriptional regulator NrdR (http://regprecise.lbl.gov/RegPrecise/regulon.jsp?regulon_id=7297) in eight Thermotogae. Finally, the 3’-terminus of *nrdG* in the *T*. *maritima* genome overlaps with the 5’-terminus of *nrdD* by 35 base pairs. Such overlapping genes are commonly taken as strong indication of co-regulation [44] Taken together, this evidence indicates that the anaerobic RNR system is enzymatically active and highly relevant for the viability of *Thermotogales*.

### tmNrdD in an evolutionary context

Two parallel subclassifications of class III RNRs have recently been presented, one in RNRdb2 (http://rnrdb.pfitmap.org; unpublished, developed by two of us (BMS and DL)), the other by Wei et al. [23]. The former identifies six subclasses (NrdDa, c, d, f, h and i), while the latter identifies three subtypes (NrdD1-3). The subclassification in RNRdb2 is based on phylogenetic evidence (in ref. [43] and unpublished) and aims to identify potentially monophyletic groups of sequences–i.e. all sequences sharing a common ancestor–while Wei et al.'s subclassification is based on chemically interesting amino acid patterns in the sequences. NrdD2 appears defined by the presence of a third Cys residue and a Glu in the active site; some NrdD2 also have a Met in the active site. NrdD3 has the third Cys but not the Glu. NrdD1 lacks all of these three amino acids (ref. [23], Fig 7). The phylogeny by Wei et al. suggests that the NrdD2 has evolved at least twice, since it forms two clades under all possible rootings of the tree. The NrdD1 subtype in Wei et al.'s classification contains the three best-studied NrdDs, from *E*. *coli*, *L*. *lactis* and bacteriophage T4. In RNRdb2 these are in the NrdDc subclass, but NrdD1 also contains methanogens from the NrdDa subclass in RNRdb2. Similarly, NrdD2 contains sequences from several RNRdb2 subclasses: NrdDh (e.g. *T*. *maritima* and *N*.*bacilliformes*), NrdDf (e.g. *Candidatus* Korarchaeum cryptofilum), NrdDi (e.g. *Ignicoccus hospitalis*, *Thermococcus gammatolerans* and *Pyrobaculum islandicum*), NrdDd (e.g. *Chlorobium limicola*, *Rhodobacter capsulatus* and *Desulfovibrio desulfuricans*), NrdDa (e.g. *Aminomonas paucivorans*). The NrdD3 subtype appears only to contain NrdDas, although some NrdDas are not part of NrdD3 but of NrdD2. The considerable differences between the two subclassifications reflect the different philosophies behind them. While the purpose of the subclassification in RNRdb2 is to provide an evolutionary framework to reach a deeper understanding of biochemistry, the subclassification by Wei et al. describes the biochemistry and maps it onto a phylogeny to decipher the potential evolutionary background to the three subtypes. By basing the subclassification on the presence or absence of only three amino acids, the analysis by Wei et al. risks missing information in the sequence that may prove important for further biochemical characterization.Although the two subclassifications are profoundly different, we can agree that tmNrdD represents the first solved structure from a new subclass (NrdDh in our preferred terminology) that is different in important and interesting ways from the NrdDs studied earlier (*E*. *coli*, *L*. *lactis* and bacteriophage T4 NrdDc). Moreover, the diversity within the NrdDh subclass is substantial, with four clearly identifiable groups, NrdDh1-4, and some as yet unclassified phage sequences (Fig 7 and S6 Fig). In particular we note an insertion of 14 amino acids in the *T*. *maritima* sequence compared with NrdDh3 and 4, a region of the sequence that is not well ordered in the structure and creates some difficulty in identification of the amino acid in NrdDh2 and 3 that is homologous to the Ile at the canonical radical Cys position in NrdDh1. Despite the divergences within the subclass, we find that important motifs are conserved. All sequences share the SCCR motif N-terminal of the Ile, except for the MCCR variant in NrdDh4 (Fig 7 and S6 Fig). The SCCR motif is not found in any other NrdDs except NrdDh, indicating functional specialization.

### The tmNrdD structure is consistent with a novel newly-proposed thiosulphuranyl radical

Recently a novel thiosulphuranyl radical in the class III RNR mechanism was proposed based on work with *E*. *coli* NrdD [48]. This radical involves the covalent crosslinking of the conserved Cys that participates in the reductive half-reaction (Cys79 in T4, Cys175 in *E*. *coli* and Cys125 in tmNrdD) with the S atom of a Met residue in the finger loop. This is stereochemically feasible, since the S atoms of the Cys and Met residues are only 4.4 Å from each other in the T4NrdD structure and 3.3 Å in a homology model of the *E*. *coli* enzyme (S7 Fig). Interestingly, although the finger loop sequence is very different in tmNrdD and traverses the inside of the α-β barrel in the opposite direction to T4NrdD (S3 Fig), Met356, three residues upstream of Ile359, has two side chain conformations of which one is oriented towards Cys125, bringing the S atoms to 4.1 Å and 4.4 Å from each other in monomers A and B respectively. (S7 Fig). Therefore the tmNrdD structure is consistent with the proposed reaction mechanism. However Met356 is only observed in a minority of NrdDh1 sequences, thus other variants of the reaction mechanism most likely apply to NrdDh1 enzymes lacking Met as well as groups NrdDh2-4.

### What is the active conformation of tmNrdD?

Wei and coworkers recently demonstrated that thioredoxin can be used as reductant by the class III RNR from *N*. *bacilliformis*, nbNrdD [23], in contrast to formate for other previously characterized NrdDs. NbNrdD belongs to group NrdDh3 in our phylogenetic analysis. The attribution of reduction activity to a protein is consistent with our finding for *T*. *maritima* that the essential component is found in the retentate when cell extracts are filtered using a 10 kDa cutoff. The *N*. *bacilliformis* study also included a crystal structure of tmNrdD, albeit without substrate or allosteric effector (PDB ID 4U3E). Similarly to our study, Wei *et al*. found the Ile-containing sequence (^358^SIGG^361^) at the tip of the finger loop but interpreted this as a functionally irrelevant artifact caused by proteolytic cleavage of the polypeptide in the region of the flexible SCCR motif and insertion of the “wrong” sequence into the β-barrel. A “hybrid” structural model for tmNrdD was presented in which the SCCR motif was manually placed in the active site based on its position in the T4 enzyme structure [9] and this model was used to interpret the data from the *N*. *bacilliformis* NrdD, to which tmNrdD has 30% sequence identity.

As for tmNrdD, mutations in both of the cysteine residues in the SCCR motif of nbNrdD produced enzymes inactive in CTP production [23]. Mutation of the second Cys to Ala resulted in production of cytosine instead of dCTP. By analogy to earlier experiments on the class I R1 protein from *E*. *coli* involving mutation of Cys462, the second cysteine in the disulfide-forming redox pair, which also resulted in the production of cytosine [49], this experiment provided evidence that the cysteines of the SCCR motif may be localized in the active site at some point in the reaction cycle. Our mutations of the equivalent residues in tmNrdD also demonstrate that they are essential for tmNrdD activity. Nevertheless the unexpected presence of the sequence SIGG (equivalent to SLVG in nbNrdD) in a stable, well-ordered and energetically favorable conformation in the interior of the 10-stranded RNR barrel is intriguing and cannot easily be dismissed as an artifact. Furthermore the crystal structure of the dATP/CTP complex shows that the SIGG motif is compatible with substrate binding. Indeed stabilizing interactions are made between substrate ribose hydroxyl groups and main chain atoms of the finger loop, whose conformation is dependent on its local sequence. Further arguments can be made against the idea that the crystal structure is an artifact. Firstly, in the work by Wei et al., dissolved unwashed crystals of the type used to determine the crystal structure showed enrichment of proteolytically cleaved protein. The electron density for 4U3E and our study both show 100% occupancy of the SIGG motif, with identical structures. We have analyzed carefully washed and redissolved crystals of tmNrdD using SDS-PAGE and do not observe any proteolytic cleavage (S8 Fig), thus the presence of the Ile-containing loop in our structure is not due to proteolytic degradation of the protein sample. Secondly, the protein in our study was purified using a very different protocol to that of Wei and coworkers, including heat treatment at 75°C, but we nevertheless see the same three-dimensional structure. It is not impossible that the observed conformation arises from crystallizing a thermophilic protein at room temperature, but the fact that we heated the protein to 75°C during purification speaks against this possibility.

Thus it seems likely that the structure of tmNrdD represents a conformation of the finger loop relevant for the reactive cycle. The SIGG motif could represent a stable “resting” form of the enzyme from which a large and unprecedented conformational change occurs upon generation of an active enzyme in order to place the SCCR motif in the active site. Alternatively, as mentioned above, the *T*. *maritima* cell extracts most likely contain a Trx-like component needed to shuttle the electrons delivered by the photoreduced deazaflavin to the active site. Therefore, by analogy to the cysteines near the C-terminus of the class I R1 proteins [50], the SCCR motif could be essential in this transfer. However this would most likely require an in-trans interaction between NrdD dimers, as the SCCR motif cannot reach into the active site when this is already filled by the finger loop. Finally, we found that the *E*. *coli* thioredoxin was unable to substitute for *T*. *maritima* cell extracts in activity assays, implying that there may be significant structural and mechanistic differences between *T*. *maritima* and *N*. *bacilliformis*. As noted, the proximity of Met356 to Cys125 in tmNrdD is compatible with the reaction mechanism proposed for the majority of class III RNRs, involving a thiosulphuranyl radical [48].

It is plausible that large conformational changes introduce cysteines from the SCCR motif into the active site, at least in some species. However if we consider the alternative possibility that the finger loop conformation seen in the crystal structure of tmNrdD is the one present during initiation of substrate reduction in *T*. *maritima*, how could the glycyl radical be transferred from the C-terminal loop to the substrate? The active site area lacks an obvious alternative H-bonded radical transfer pathway (Fig 5). The glycyl radical is highly stable due to spin delocalization over a planar C_α_-peptide bond system and more substituted carbon-based radicals are significantly less stable [51,52]. Ile359 is wedged between Gly621 and the substrate, with its side chain oriented towards Gly621. In principle, one of the γ-H atoms on Ile359, at 3.8 Å from Gly621, could be abstracted to generate an unstable radical on Cγ1 (black dashes in Fig 5). This might be favored by concerted abstraction of the substrate H3’, but the distance is too large (4.9 Å). In addition, as mentioned above, many other members of the NrdDh class possibly contain a Phe residue that makes this scenario less likely. Alternatively, a radical centered on C_α_ of Ile359 would be stable and is closer to H3’ (4.3 Å), but the C_α_ of Ile359 is sterically blocked from Gly621 by its own side chain (blue dashes in Fig 5). While at present the most likely scenario seems to be a conformational change that introduced the SCCR motif into the active site, further mutational analysis of residues in the finger loop of tmNrdD and studies of other NrdDh family members are required to establish the role of the finger loop in these enzymes.

## Conclusions

The structure of *T*. *maritima* NrdD shows for the first time how triphosphate substrates can bind to an RNR and additionally provides a surprising example of a ribonucleotide reductase in which a cysteine residue for initiation of radical chemistry on the substrate is not pre-positioned at the tip of the finger loop in the active site in the enzyme as purified and crystallized, as found independently by two research groups. A cysteine residue may arrive there through conformational changes of a magnitude unprecedented for RNRs, but this is not yet completely established for the *T*. *maritima* enzyme, and further experiments are clearly required to resolve current ambiguities.

## Supporting Information

S1 Fig Occupancy of the active site of tmNrdD by various buffer components. a) MES; b) citrate; c) glycerol; d) superposition of all; e) details of the interaction of MES with tmNrdD. In each panel a 2|Fo|-mD|Fc| map is shown for the ligand, contoured at 1.0 σ and in panels a)-d) the structure of bound CTP in the dATP/CTP complex is shown for comparison. In panel e) all water molecules forming a network between MES and the protein are shown as red spheres and relevant hydrogen bonds as dotted lines.
(TIF)

S2 Fig The Zn binding site in tmNrdD. a) Structure of the Zn binding site on the surface of tmNrdD; b) the Zn site in NrdD from bacteriophage T4, for comparison. The Zn ions are shown as a grey spheres. The entire C-terminal domain containing the Zn site and the loop housing the glycyl radical (G621 in tmNrdD and G580 in T4NrdD) is coloured orange. The other monomer of each dimer (on the left) is coloured in a deeper shade in each panel. Allosteric effector dATP is shown in stick representation for both proteins and the substrate CTP is also shown as sticks for tmNrdD. In panel a) an anomalous difference map for Zn is shown, contoured at 10.0 σ. The data for this map were collected at a wavelength just below that of the Zn K edge (1.2822 Å, S1 Table).
(TIF)

S3 Fig Stereo figure showing details of the interactions of the finger loops in a) tmNrdD and b) T4NrdD with the insides of their respective 10-stranded α-β barrels. Carbon atoms in the finger loops are coloured from blue at the N-terminal end to red at the C-terminal end to emphasise the different directions of travel of the loops in the two structures. Hydrogen bonding interactions from the finger loop to nearby protein residues and water molecules are shown as dotted lines. Where the hydrogen bonds do not terminate in a depicted protein side chain, this means that the bond is to a protein main chain atom, but for clarity these are not shown.
(TIF)

S4 Fig Electron density for the CTP substrate in the dATP/CTP complex of tmNrdD and the surrounding protein residues. A 2|Fo|-mD|Fc| omit map is shown contoured at 1.0 σ. The map was generated by omitting the CTP and surrounding water molecules from the model, adding random shifts with a rms value of 0.5 Å to the coordinates and three macrocycles of refinement in phenix.refine.
(TIF)

S5 Fig EPR spectrum at 20 K of the glycyl radical of activated TmNrdD a) in H_2_O; b) in D_2_O. The glycyl radical content, estimated using a Cu^2+^ standard, is about 0.15 Gly° per monomer. Experimental details are given in S1 Text.
(TIF)

S6 Fig Phylogenetic network (NeighborNet) of representative sequences from the NrdDh diversity. The repertoire of encoded RNRs other than NrdDh in each organism is indicated with coloured circles, one per class found at least once in the same genome, except for class III where a circle shows the presence of another subclass than IIIh. Blue circle: class I; green circle: class II; red circle: other class III. The groups NrdDh1-4 are shown in yellow with sequence motifs as in Fig 5.
(TIF)

S7 Fig Proximity of the cysteine participating in the reductive half-reaction to a Met residue in the finger loop of NrdDs from three species: a) bacteriophage T4 [9]; b) *E*. *coli* (a homology model from the Modweb server [55] based on the T4 structure); c) *T*. *maritima* (this work). (TIF)

S8 Fig Coomassie-stained SDS-PAGE gel of tmNrdD and dissolved crystals of tmNrdD. Lane 1: dissolved, washed crystals; lane 2: protein suspended over its own buffer as reservoir, for the same length of time as it took for the crystals used in lane 1 to appear; lane 3: protein taken directly from storage at -80°C.
(PDF)

S1 Table Statistics for the X-ray crystallography data sets. Friedel pairs are treated as the same reflection, except for the peak, inflection, remote, dATP #2 and dATP #3 datasets where they are treated as different reflections. The space group was P2_1_ for all datasets. Values in parentheses are for the highest resolution bins.
(DOCX)

S2 Table Refinement and model quality statistics for five tmNrdD structures deposited in the PDB. Numbers in parentheses are for the highest resolution bins, unless otherwise noted.
(DOCX)

S3 Table Structural comparison of all known large subunits of ribonucleotide reductases. Unique ribonucleotide reductase catalytic subunits were selected based on DALI [53] results using 4COI (chain A) as a search structure. The structures were pairwise superimposed using the SSM algorithm [54] in SUPERPOSE from the CCP4 suite. RMSD values for equivalent Cα positions are listed in Å and in parenthesis the number of superimposed CA positions is given. 4COI_A: *Thermotoga maritima* class III (607 Cα), 1H7B_A: Bacteriophage T4 class III (534 Cα), 3O0O_B: *Thermotoga maritima* class II (626 Cα), 3HNC_A: *Homo sapiens* class Ia (714 Cα), 1L1L_B: *Lactobacillus leichmannii* class II (717 Cα), 2CVX_A: *Saccharomyces cerevisiae* class Ia (664 Cα), 1PEU_A: *Salmonella typhimurium* class Ib (692 CA) and 1RLR_A: *Escherichia coli* class Ia (737 Cα).
(DOCX)

S1 Text Detailed experimental procedures. (DOCX)

## References

[1] Eklund H, Uhlin U, Färnegårdh M, Logan DT, Nordlund P (2001) Structure and function of the radical enzyme ribonucleotide reductase. Prog Biophys Mol Biol 77: 177–268. 11796141 10.1016/s0079-6107(01)00014-1

[2] Petersson L, Gräslund A, Ehrenberg A, Sjöberg BM, Reichard P (1980) The iron center in ribonucleotide reductase from *Escherichia coli* . J Biol Chem 255: 6706–6712. 6248531

[3] Tamao Y, Blakley RL (1973) Direct spectrophotometric observation of an intermediate formed from deoxyadenosylcobalamin in ribonucleotide reduction. Biochemistry 12: 24–34. 4566928 10.1021/bi00725a005

[4] Larsson KM, Logan DT, Nordlund P (2010) Structural basis for adenosylcobalamin activation in AdoCbl-dependent ribonucleotide reductases. ACS Chem Biol 5: 933–942. 10.1021/cb1000845 20672854

[5] Sun X, Eliasson R, Pontis E, Andersson J, Buist G, et al (1995) Generation of the glycyl radical of the anaerobic *Escherichia coli* ribonucleotide reductase requires a specific activating enzyme. J Biol Chem 270: 2443–2446. 7852304 10.1074/jbc.270.6.2443

[6] Ollagnier S, Mulliez E, Schmidt PP, Eliasson R, Gaillard J, et al (1997) Activation of the anaerobic ribonucleotide reductase from *Escherichia coli*. The essential role of the iron-sulfur center for S- adenosylmethionine reduction. J Biol Chem 272: 24216–24223. 9305874 10.1074/jbc.272.39.24216

[7] Sun X, Ollagnier S, Schmidt PP, Atta M, Mulliez E, et al (1996) The free radical of the anaerobic ribonucleotide reductase from *Escherichia coli* is at glycine 681. J Biol Chem 271: 6827–6831. 8636106

[8] Young P, Andersson J, Sahlin M, Sjöberg BM (1996) Bacteriophage T4 anaerobic ribonucleotide reductase contains a stable glycyl radical at position 580. J Biol Chem 271: 20770–20775. 8702830 10.1074/jbc.271.34.20770

[9] Logan DT, Andersson J, Sjoberg BM, Nordlund P (1999) A glycyl radical site in the crystal structure of a class III ribonucleotide reductase. Science 283: 1499–1504. 10066165 10.1126/science.283.5407.1499

[10] Torrents E, Eliasson R, Wolpher H, Gräslund A, Reichard P (2001) The anaerobic ribonucleotide reductase from *Lactococcus lactis*. Interactions between the two proteins NrdD and NrdG. J Biol Chem 276: 33488–33494. 11427536 10.1074/jbc.M103743200

[11] Luttringer F, Mulliez E, Dublet B, Lemaire D, Fontecave M (2009) The Zn center of the anaerobic ribonucleotide reductase from *E*. *coli* . J Biol Inorg Chem 14: 923–933. 10.1007/s00775-009-0505-9 19381696

[12] Uhlin U, Eklund H (1994) Structure of ribonucleotide reductase protein R1. Nature 370: 533–539. 8052308 10.1038/370533a0

[13] Sintchak MD, Arjara G, Kellogg BA, Stubbe J, Drennan CL (2002) The crystal structure of class II ribonucleotide reductase reveals how an allosterically regulated monomer mimics a dimer. Nat Struct Biol 9: 293–300. 11875520 10.1038/nsb774

[14] Larsson KM, Jordan A, Eliasson R, Reichard P, Logan DT, et al (2004) Structural mechanism of allosteric substrate specificity regulation in a ribonucleotide reductase. Nat Struct Mol Biol 11: 1142–1149. 15475969 10.1038/nsmb838

[15] Stubbe J, Ator M, Krenitsky T (1983) Mechanism of ribonucleoside diphosphate reductase from *Escherichia coli*. Evidence for 3'-C—H bond cleavage. J Biol Chem 258: 1625–1631. 6337142

[16] Becker A, Fritz-Wolf K, Kabsch W, Knappe J, Schultz S, et al (1999) Structure and mechanism of the glycyl radical enzyme pyruvate formate lyase. Nat Struct Biol 6: 969–975. 10504733 10.1038/13341

[17] O'Brien JR, Raynaud C, Croux C, Girbal L, Soucaille P, et al (2004) Insight into the mechanism of the B12-independent glycerol dehydratase from *Clostridium butyricum*: preliminary biochemical and structural characterization. Biochemistry 43: 4635–4645. 15096031 10.1021/bi035930k

[18] Martins BM, Blaser M, Feliks M, Ullmann GM, Buckel W, et al (2011) Structural basis for a Kolbe-type decarboxylation catalyzed by a glycyl radical enzyme. J Am Chem Soc 133: 14666–14674. 10.1021/ja203344x 21823587

[19] Funk MA, Judd ET, Marsh EN, Elliott SJ, Drennan CL (2014) Structures of benzylsuccinate synthase elucidate roles of accessory subunits in glycyl radical enzyme activation and activity. Proc Natl Acad Sci U S A 111: 10161–10166. 10.1073/pnas.1405983111 24982148 PMC4104874

[20] Thelander L (1974) Reaction mechanism of ribonucleoside diphosphate reductase from *Escherichia coli*. Oxidation-reduction-active disulfides in the B1 subunit. J Biol Chem 249: 4858–4862. 4152559

[21] Eriksson M, Uhlin U, Ramaswamy S, Ekberg M, Regnström K, et al (1997) Binding of allosteric effectors to ribonucleotide reductase protein R1: reduction of active-site cysteines promotes substrate binding. Structure 5: 1077–1092. 9309223 10.1016/s0969-2126(97)00259-1

[22] Mulliez E, Ollagnier S, Fontecave M, Eliasson R, Reichard P (1995) Formate is the hydrogen donor for the anaerobic ribonucleotide reductase from *Escherichia coli* . Proc Natl Acad Sci USA 92: 8759–8762. 7568012 10.1073/pnas.92.19.8759 PMC41046

[23] Wei Y, Funk MA, Rosado LA, Baek J, Drennan CL, et al (2014) The class III ribonucleotide reductase from *Neisseria bacilliformis* can utilize thioredoxin as a reductant. Proc Natl Acad Sci U S A 111: E3756–3765. 10.1073/pnas.1414396111 25157154 PMC4246965

[24] Nordlund P, Reichard P (2006) Ribonucleotide reductases. Annu Rev Biochem 75: 681–706. 16756507 10.1146/annurev.biochem.75.103004.142443

[25] Nurizzo D, Mairs T, Guijarro M, Rey V, Meyer J, et al (2006) The ID23-1 structural biology beamline at the ESRF. Journal of Synchrotron Radiation 13: 227–238. 16645249 10.1107/S0909049506004341

[26] Ursby T, Unge J, Appio R, Logan DT, Fredslund F, et al (2013) The macromolecular crystallography beamline I911-3 at the MAX IV laboratory. Journal of Synchrotron Radiation 20: 648–653. 10.1107/S0909049513011734 23765310 PMC3943556

[27] Panjikar S, Parthasarathy V, Lamzin VS, Weiss MS, Tucker PA (2005) Auto-Rickshaw: an automated crystal structure determination platform as an efficient tool for the validation of an X-ray diffraction experiment. Acta Crystallographica Section D-Biological Crystallography 61: 449–457. 10.1107/S0907444905001307 15805600

[28] Logan DT (2008) The anaerobic ribonucleotide reductases: recent progress in understanding In: Andersson KK, editor. Ribonucleotide reductase. Hauppauge, NY: Nova Science Publishers pp. 185–208.

[29] Larsson K-M, Andersson J, Sjöberg B-M, Nordlund P, Logan DT (2001) Structural basis for allosteric substrate specificity regulation in anaerobic ribonucleotide reductases. Structure 9: 739–750. 11587648 10.1016/s0969-2126(01)00627-x

[30] Logan DT, Mulliez E, Larsson KM, Bodevin S, Atta M, et al (2003) A metal-binding site in the catalytic subunit of anaerobic ribonucleotide reductase. Proc Natl Acad Sci U S A 100: 3826–3831. 12655046 10.1073/pnas.0736456100 PMC153006

[31] Lundin D, Torrents E, Poole AM, Sjöberg BM (2009) RNRdb, a curated database of the universal enzyme family ribonucleotide reductase, reveals a high level of misannotation in sequences deposited to Genbank. BMC Genomics 10: 589 10.1186/1471-2164-10-589 19995434 PMC2795772

[32] Becker A, Kabsch W (2002) X-ray structure of pyruvate formate lyase in complex with pyruvate and CoA. How the enzyme uses the Cys-418 thiyl radical for pyruvate cleavage. J Biol Chem 277: 40036–40042. 12163496 10.1074/jbc.M205821200

[33] Vey JL, Yang J, Li M, Broderick WE, Broderick JB, et al (2008) Structural basis for glycyl radical formation by pyruvate formate-lyase activating enzyme. Proc Natl Acad Sci U S A 105: 16137–16141. 10.1073/pnas.0806640105 18852451 PMC2571006

[34] Uppsten M, Färnegårdh M, Jordan A, Eliasson R, Eklund H, et al (2003) Structure of the large subunit of class Ib ribonucleotide reductase from Salmonella typhimurium and its complexes with allosteric effectors. J Mol Biol 330: 87–97. 12818204 10.1016/s0022-2836(03)00538-2

[35] Xu H, Faber C, Uchiki T, Fairman JW, Racca J, et al (2006) Structures of eukaryotic ribonucleotide reductase I provide insights into dNTP regulation. Proc Natl Acad Sci U S A 103: 4022–4027. 16537479 10.1073/pnas.0600443103 PMC1389704

[36] Ahmad MF, Kaushal PS, Wan Q, Wijerathna SR, An X, et al (2012) Role of arginine 293 and glutamine 288 in communication between catalytic and allosteric sites in yeast ribonucleotide reductase. J Mol Biol 419: 315–329. 10.1016/j.jmb.2012.03.014 22465672 PMC3589814

[37] Fairman JW, Wijerathna SR, Ahmad MF, Xu H, Nakano R, et al (2011) Structural basis for allosteric regulation of human ribonucleotide reductase by nucleotide-induced oligomerization. Nat Struct Mol Biol 18: 316–322. 10.1038/nsmb.2007 21336276 PMC3101628

[38] Andersson J, Bodevin S, Westman M, Sahlin M, Sjöberg B-M (2001) Two active site asparagines are essential for the reaction mechanism of the class III anaerobic ribonucleotide reductase from bacteriophage T4. J Biol Chem 276: 40457–40463. 11526118 10.1074/jbc.M106863200

[39] Mulliez E, Fontecave M, Gaillard J, Reichard P (1993) An iron-sulfur center and a free radical in the active anaerobic ribonucleotide reductase of *Escherichia coli* . J Biol Chem 268: 2296–2299. 8381402

[40] Torrents E, Buist G, Liu A, Eliasson R, Kok J, et al (2000) The anaerobic (class III) ribonucleotide reductase from *Lactococcus lactis*. Catalytic properties and allosteric regulation of the pure enzyme system. J Biol Chem 275: 2463–2471. 10644700 10.1074/jbc.275.4.2463

[41] Wagner AF, Frey M, Neugebauer FA, Schafer W, Knappe J (1992) The free radical in pyruvate formate-lyase is located on glycine-734. Proc Natl Acad Sci U S A 89: 996–1000. 1310545 10.1073/pnas.89.3.996 PMC48372

[42] Huber R, Langworthy TA, Konig H, Thomm M, Woese CR, et al (1986) *Thermotoga maritima* sp. nov. represents a new genus of unique extremely thermophilic eubacteria growing up to 90°C. Archives of Microbiology 144: 324–333.

[43] Johansson R, Torrents E, Lundin D, Sprenger J, Sahlin M, et al (2010) High-resolution crystal structures of the flavoprotein NrdI in oxidized and reduced states—an unusual flavodoxin. FEBS J 277: 4265–4277. 10.1111/j.1742-4658.2010.07815.x 20831589

[44] Latif H, Lerman JA, Portnoy VA, Tarasova Y, Nagarajan H, et al (2013) The genome organization of *Thermotoga maritima* reflects its lifestyle. PLoS Genet 9: e1003485 10.1371/journal.pgen.1003485 23637642 PMC3636130

[45] Johnson MR, Conners SB, Montero CI, Chou CJ, Shockley KR, et al (2006) The *Thermotoga maritima* phenotype is impacted by syntrophic interaction with *Methanococcus jannaschii* in hyperthermophilic coculture. Appl Environ Microbiol 72: 811–818. 16391122 10.1128/AEM.72.1.811-818.2006 PMC1352257

[46] Montero CI, Johnson MR, Chou CJ, Conners SB, Geouge SG, et al (2007) Responses of wild-type and resistant strains of the hyperthermophilic bacterium *Thermotoga maritima* to chloramphenicol challenge. Appl Environ Microbiol 73: 5058–5065. 17557852 10.1128/AEM.00453-07 PMC1951032

[47] Novichkov PS, Laikova ON, Novichkova ES, Gelfand MS, Arkin AP, et al (2010) RegPrecise: a database of curated genomic inferences of transcriptional regulatory interactions in prokaryotes. Nucleic Acids Res 38: D111–118. 10.1093/nar/gkp894 19884135 PMC2808921

[48] Wei Y, Mathies G, Yokoyama K, Chen J, Griffin RG, et al (2014) A chemically competent thiosulfuranyl radical on the *Escherichia coli* class III ribonucleotide reductase. J Am Chem Soc 136: 9001–9013. 10.1021/ja5030194 24827372 PMC4073831

[49] Mao SS, Holler TP, Yu GX, Bollinger JM, Booker S, et al (1992) A model for the role of multiple cysteine residues involved in ribonucleotide reduction—Amazing and still confusing. Biochemistry 31: 9733–9743. 1382592 10.1021/bi00155a029

[50] Åberg A, Hahne S, Karlsson M, Larsson A, Ormö M, et al (1989) Evidence for two different classes of redox-active cysteines in ribonucleotide reductase of *Escherichia coli* . J Biol Chem 264: 12249–12252. 2663852

[51] Himo F (2000) Stability of protein-bound glycyl radical: a density functional theory study. Chemical Physics Letters 328: 270–276.

[52] Hioe J, Savasci G, Brand H, Zipse H (2011) The stability of Calpha peptide radicals: why glycyl radical enzymes? Chemistry 17: 3781–3789. 10.1002/chem.201002620 21341321

[53] Holm L, Rosenstrom P (2010) Dali server: conservation mapping in 3D. Nucleic Acids Res 38: W545–549. 10.1093/nar/gkq366 20457744 PMC2896194

[54] Krissinel E, Henrick K (2004) Secondary-structure matching (SSM), a new tool for fast protein structure alignment in three dimensions. Acta Crystallogr D Biol Crystallogr 60: 2256–2268. 15572779 10.1107/S0907444904026460

[55] Pieper U, Webb BM, Dong GQ, Schneidman-Duhovny D, Fan H, et al (2014) ModBase, a database of annotated comparative protein structure models and associated resources. Nucleic Acids Res 42: D336–346. 10.1093/nar/gkt1144 24271400 PMC3965011

