# Supplementary figures and images for: The Crystal Structure of Thermotoga maritima Class III Ribonucleotide Reductase Lacks a Radical Cysteine Pre-Positioned in the Active Site
Source: PLoS One. 2015 Jul 6;10(7):e0128199. doi: 10.1371/journal.pone.0128199 (PMC4493059; doi:10.1371/journal.pone.0128199)

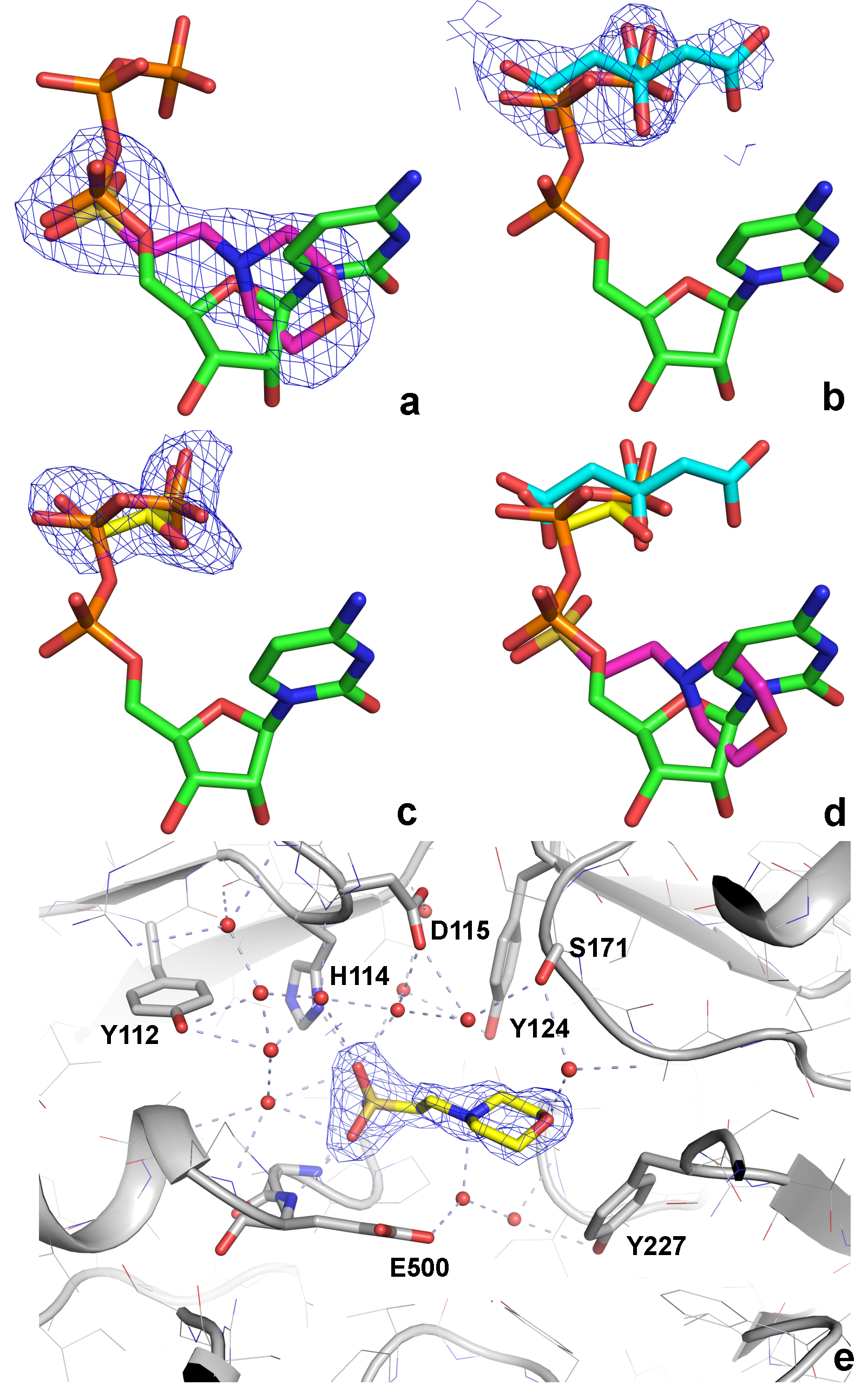

Supplement: S1 Fig — a) MES; b) citrate; c) glycerol; d) superposition of all; e) details of the interaction of MES with tmNrdD. In each panel a 2|Fo|-mD|Fc| map is shown for the ligand, contoured at 1.0 σ and in panels a)-d) the structure of bound CTP in the dATP/CTP complex is shown for comparison. In panel e) all water molecules forming a network between MES and the protein are shown as red spheres and relevant hydrogen bonds as dotted lines. (TIF) [file pone.0128199.s001.tif]

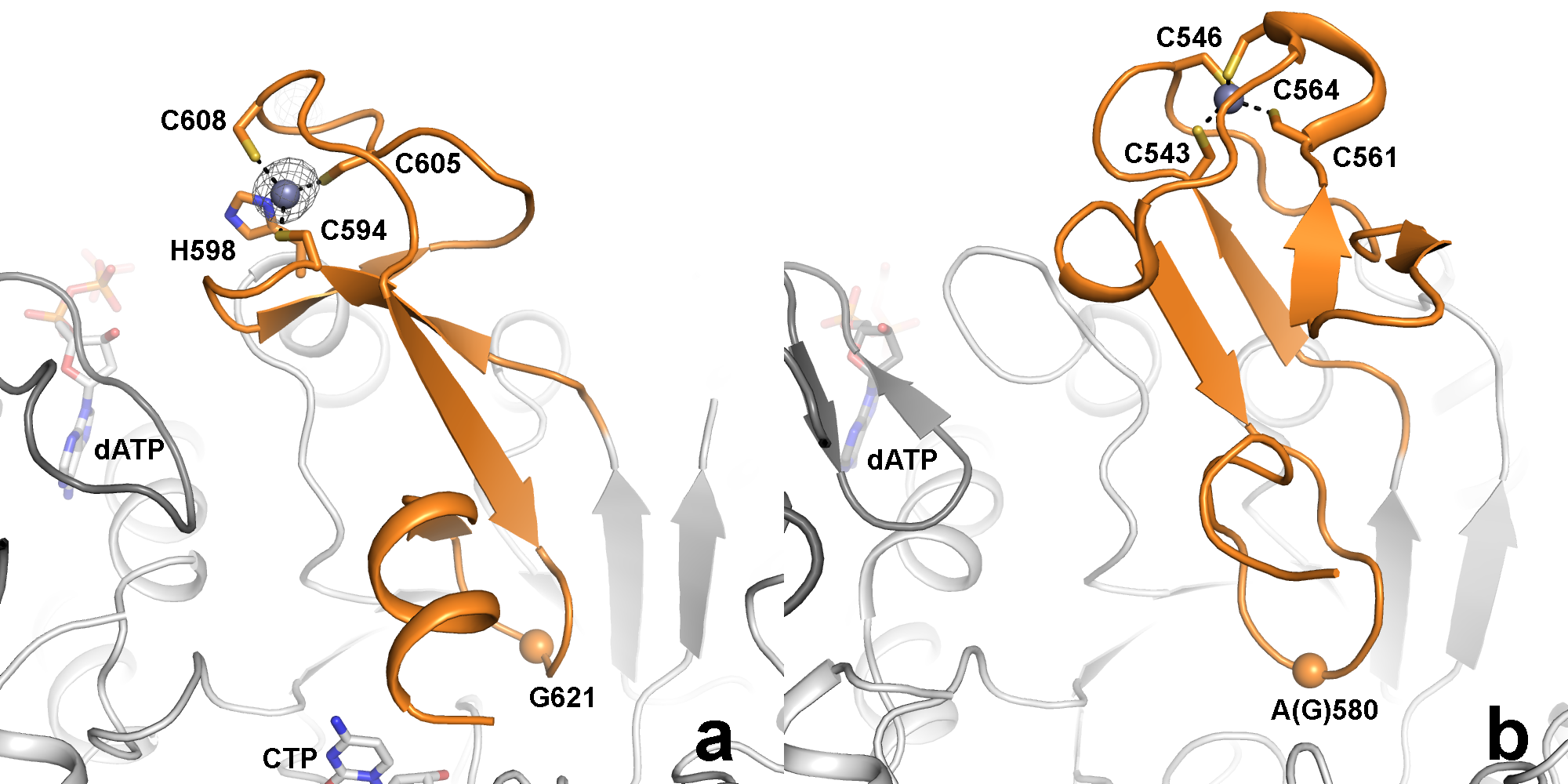

Supplement: S2 Fig — a) Structure of the Zn binding site on the surface of tmNrdD; b) the Zn site in NrdD from bacteriophage T4, for comparison. The Zn ions are shown as a grey spheres. The entire C-terminal domain containing the Zn site and the loop housing the glycyl radical (G621 in tmNrdD and G580 in T4NrdD) is coloured orange. The other monomer of each dimer (on the left) is coloured in a deeper shade in each panel. Allosteric effector dATP is shown in stick representation for both proteins and the substrate CTP is also shown as sticks for tmNrdD. In panel a) an anomalous difference map for Zn is shown, contoured at 10.0 σ. The data for this map were collected at a wavelength just below that of the Zn K edge (1.2822 Å, S1 Table). (TIF) [file pone.0128199.s002.tif]

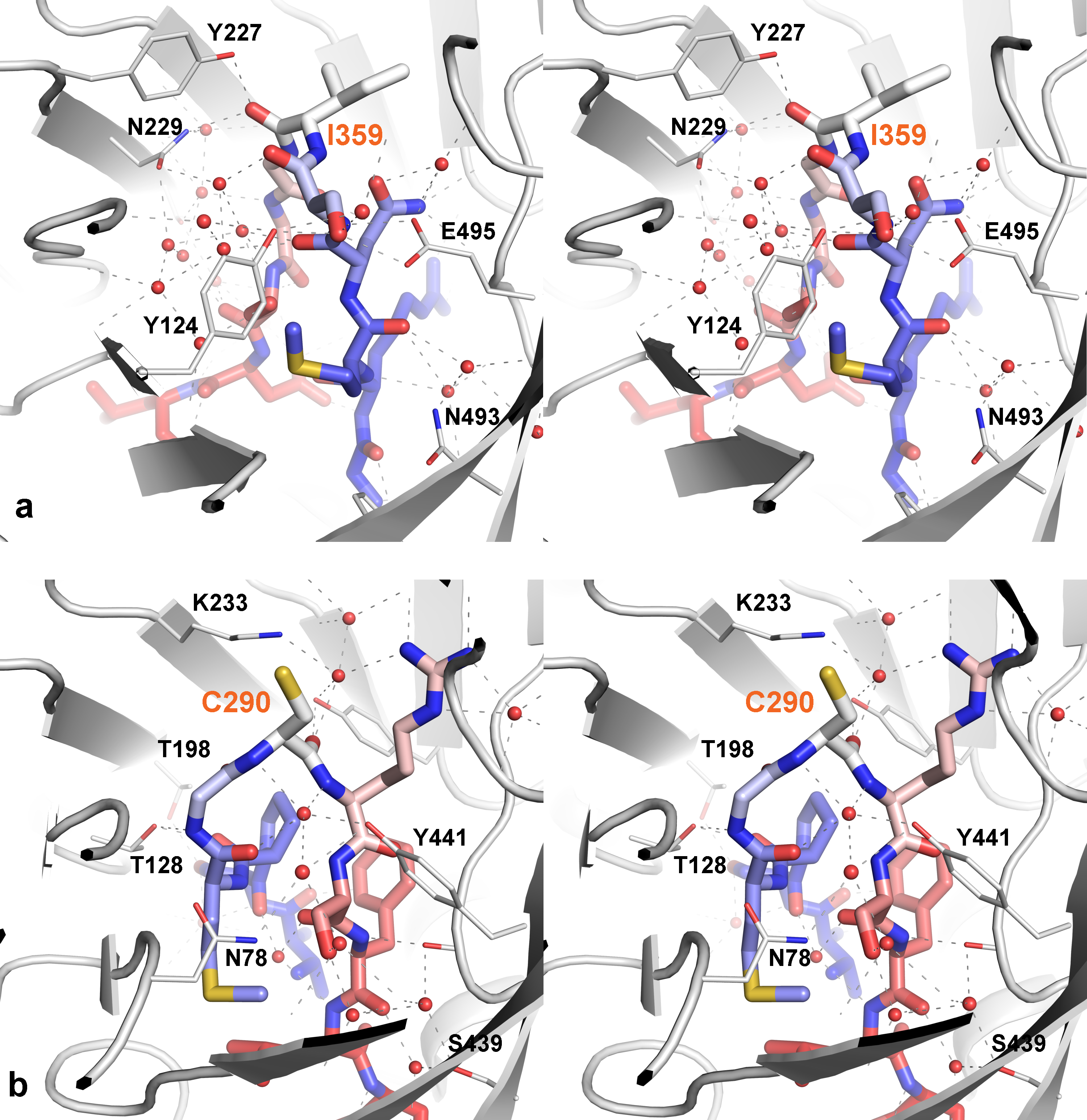

Supplement: S3 Fig — Carbon atoms in the finger loops are coloured from blue at the N-terminal end to red at the C-terminal end to emphasise the different directions of travel of the loops in the two structures. Hydrogen bonding interactions from the finger loop to nearby protein residues and water molecules are shown as dotted lines. Where the hydrogen bonds do not terminate in a depicted protein side chain, this means that the bond is to a protein main chain atom, but for clarity these are not shown. (TIF) [file pone.0128199.s003.tif]

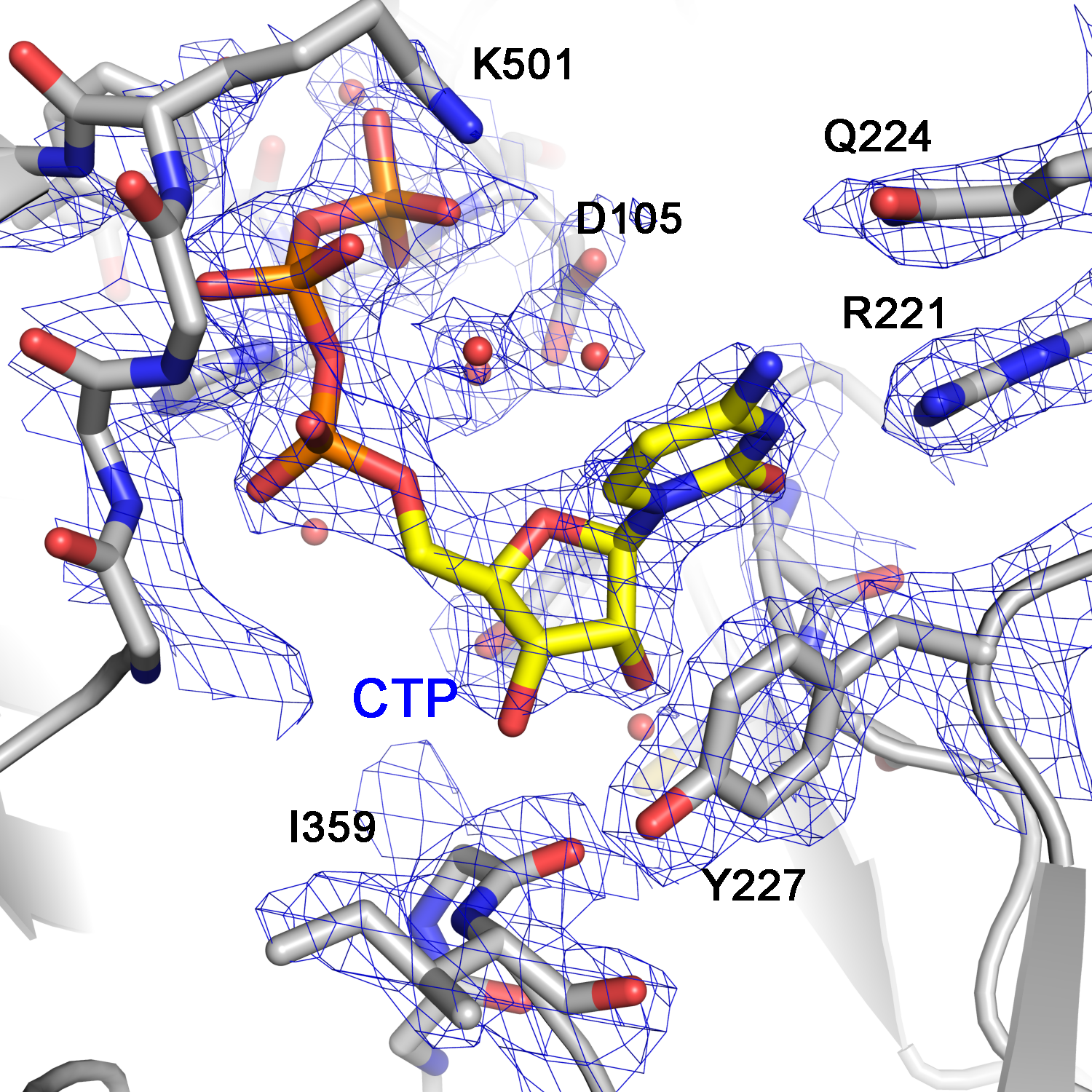

Supplement: S4 Fig — A 2|Fo|-mD|Fc| omit map is shown contoured at 1.0 σ. The map was generated by omitting the CTP and surrounding water molecules from the model, adding random shifts with a rms value of 0.5 Å to the coordinates and three macrocycles of refinement in phenix.refine. (TIF) [file pone.0128199.s004.tif]

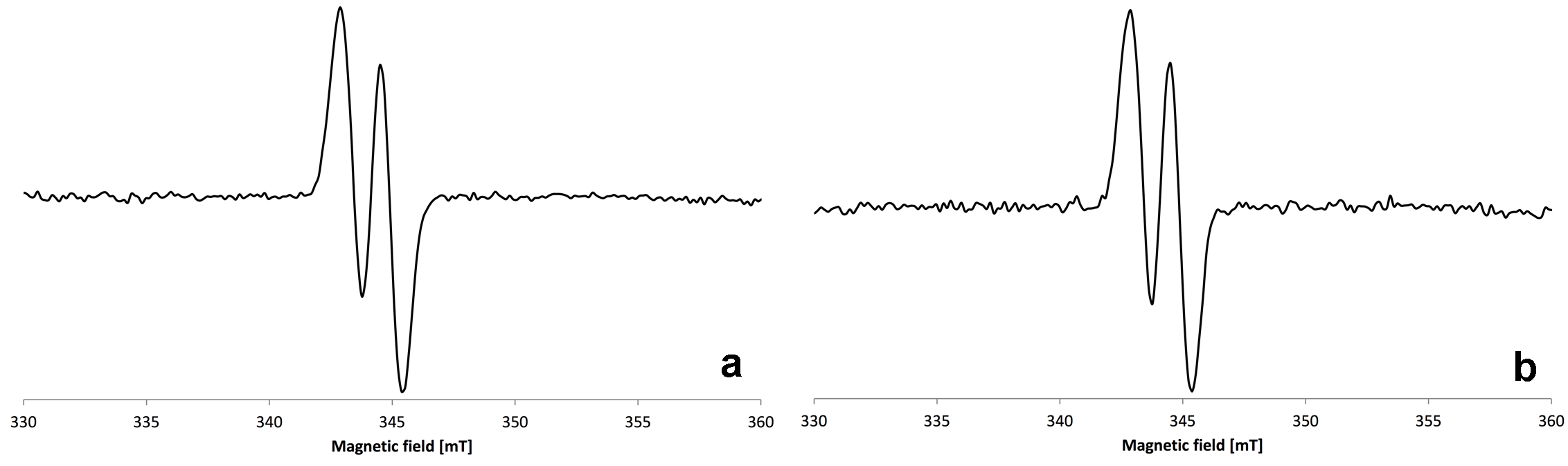

Supplement: S5 Fig — The glycyl radical content, estimated using a Cu2+ standard, is about 0.15 Gly° per monomer. Experimental details are given in S1 Text. (TIF) [file pone.0128199.s005.tif]

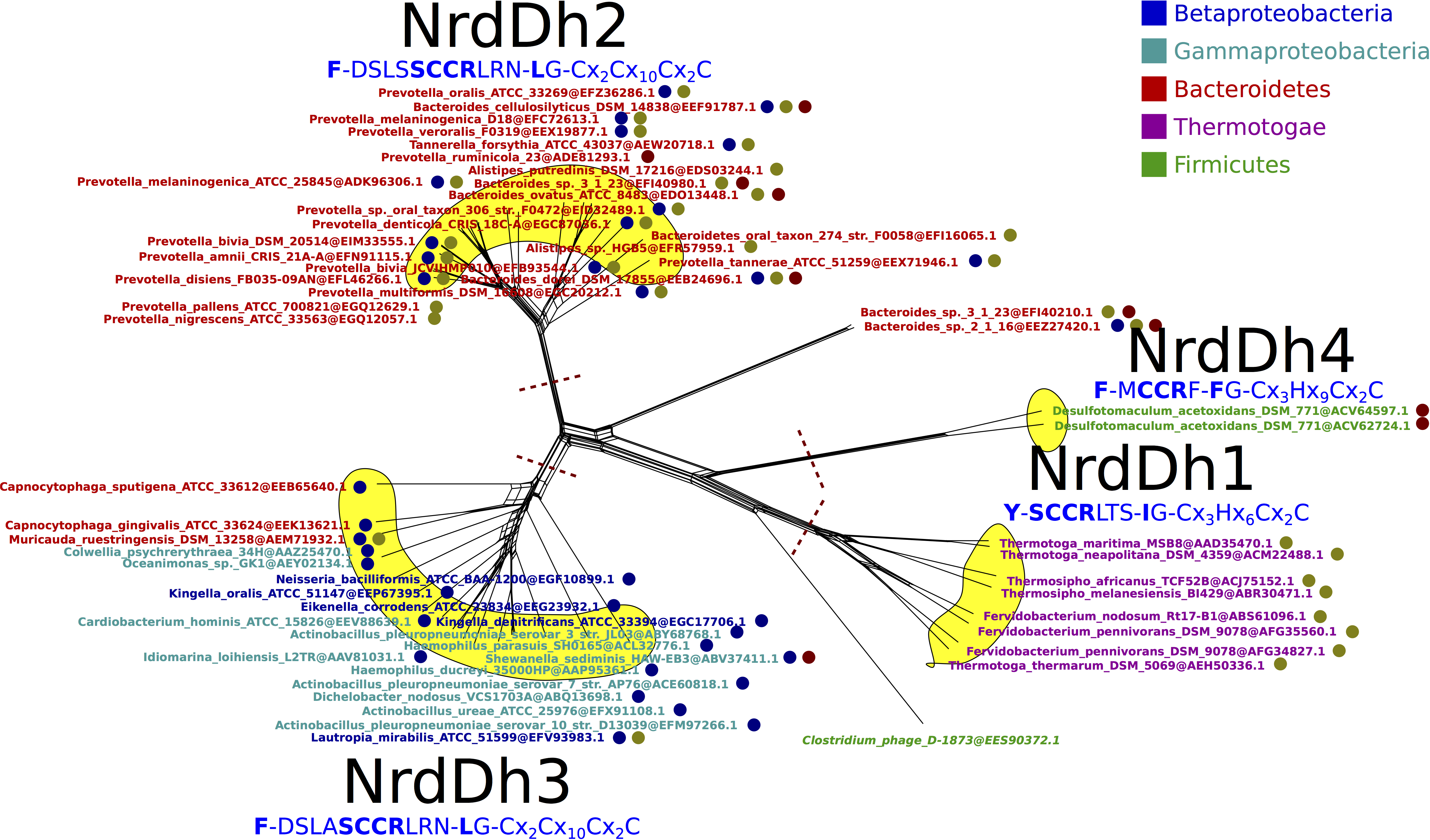

Supplement: S6 Fig — The repertoire of encoded RNRs other than NrdDh in each organism is indicated with coloured circles, one per class found at least once in the same genome, except for class III where a circle shows the presence of another subclass than IIIh. Blue circle: class I; green circle: class II; red circle: other class III. The groups NrdDh1-4 are shown in yellow with sequence motifs as in Fig 5. (TIF) [file pone.0128199.s006.tif]

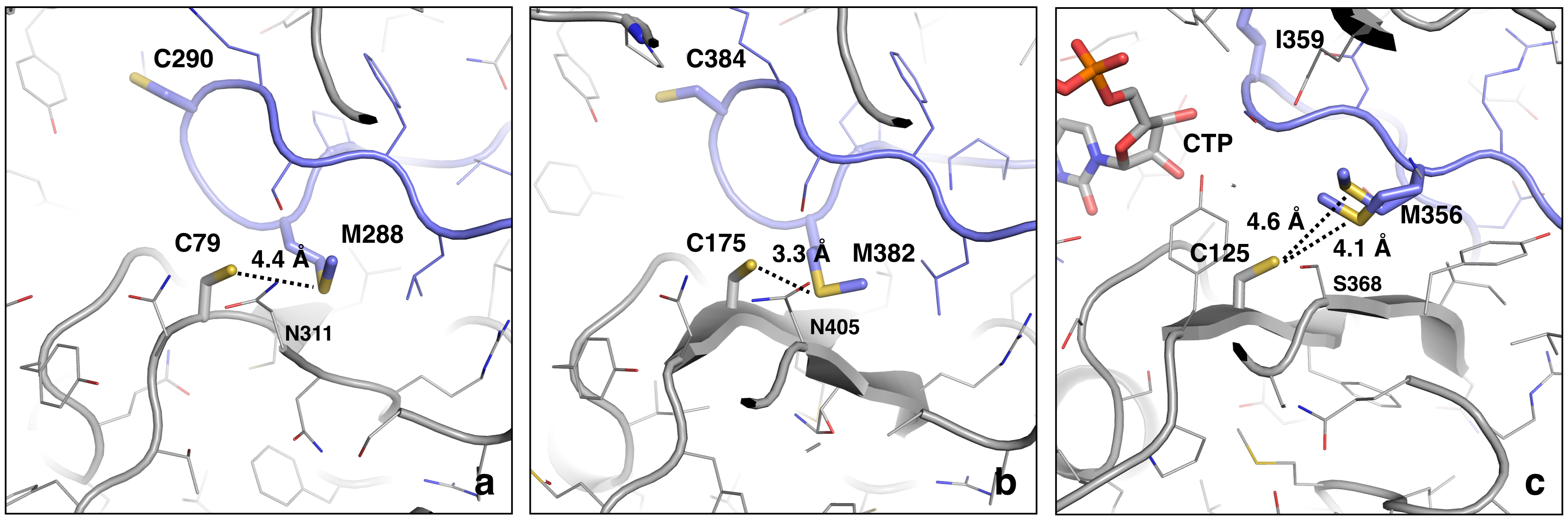

Supplement: S7 Fig — (TIF) [file pone.0128199.s007.tif]

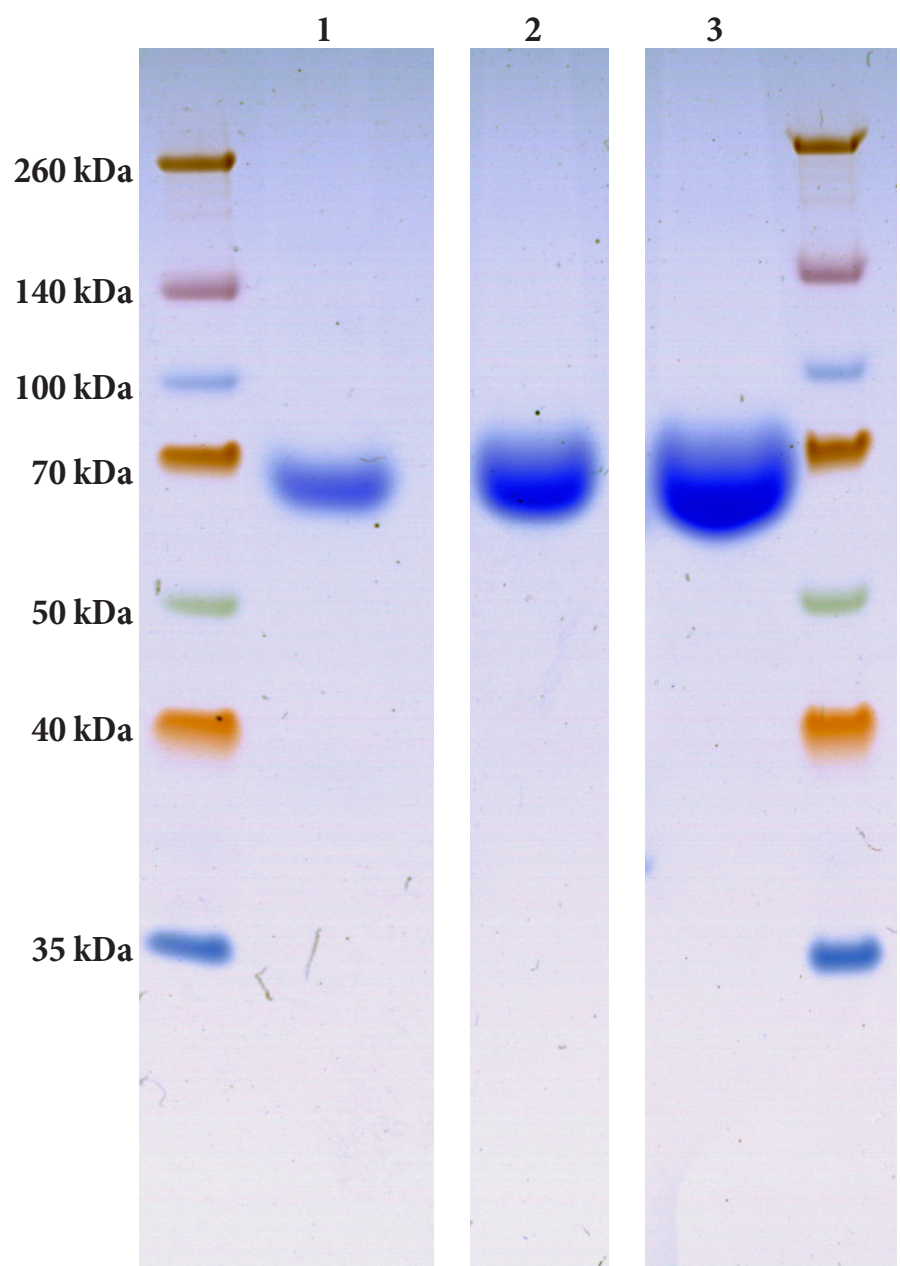

Supplement: S8 Fig — Lane 1: dissolved, washed crystals; lane 2: protein suspended over its own buffer as reservoir, for the same length of time as it took for the crystals used in lane 1 to appear; lane 3: protein taken directly from storage at -80°C. (PDF) [file pone.0128199.s008.pdf]
