# Supplementary material for: The Crystal Structure of Thermotoga maritima Class III Ribonucleotide Reductase Lacks a Radical Cysteine Pre-Positioned in the Active Site
Source: PLoS One. 2015 Jul 6;10(7):e0128199. doi: 10.1371/journal.pone.0128199 (PMC4493059; doi:10.1371/journal.pone.0128199)
Supplement: S1 Table — Friedel pairs are treated as the same reflection, except for the peak, inflection, remote, dATP #2 and dATP #3 datasets where they are treated as different reflections. The space group was P21 for all datasets. Values in parentheses are for the highest resolution bins. (DOCX) [file pone.0128199.s009.docx]

**S2 Table: Statistics for the X-ray crystallography data sets**

| **Dataset** | **Station** | **wavelength (Å)** | **unit cell parameters** | **resolution (Å)** | **unique reflections** | **completeness (%)** | **multiplicity** | **<I/ó(I)>** | **CC_1/2_** | **R_merge_(I) (%)** | **R_meas_ (%)** | **anomalous correlation coefficient (%)** | **SIGANO** |
| --- | --- | --- | --- | --- | --- | --- | --- | --- | --- | --- | --- | --- | --- |
| **Peak** | ID23-1 | 0.9792 | a = 79 Å  b = 92 Å  c = 87 Å  β = 112° | 39.1 – 2.50  (2.65 – 2.50) | 77 652  (12 386) | 99.4  (98.4) | 3.8  (3.8) | 17.2 (4.5) | 99.8 (96.0) | 5.6 (26.1) | 6.5  (30.4) | 66 (19) | 1.704 (0.832) |
| **Inflection** |  | 0.9794 |  |  | 77 613  (12 352) | 99.3  (98.1) | 3.8  (3.8) | 17.1 (4.5) | 99.8 (95.9) | 5.6 (26.3) | 6.5 (30.7) | 47 (9) | 1.301 (0.756) |
| **Remote** |  | 0.9768 |  |  | 77 651  (12 381) | 99.4  (98.4) | 3.8  (3.8) | 17.1 (4.0) | 99.8 (94.5) | 5.8  (30.4) | 6.8 (35.5) | 42 (4) | 1.233 (0.732) |
| **Native**  **(citrate, glycerol)** | I911-2 | 1.0391 | a = 78 Å  b = 99 Å  c = 87 Å  β = 112 ° | 24.3 – 1.94  (2.06 – 1.94) | 87 892  (13 445) | 97.4  (93.4) | 3.7  (3.6) | 11.3 (2.1) | 99.8 (78.9) | 7.8 (67.7) | 9.2 (79.4) | – | – |
| **Native**  **(MES)** | I911-3 | 1.0097 | a = 80 Å  b = 96 Å  c = 89 Å  β = 113 ° | 29.4 – 1.89  (2.00 – 1.89) | 100 224  (15 384) | 98.8  (94.5) | 7.2  (5.8) | 19.8 (3.2) | 99.9 (92.0) | 5.8 (48.3) | 6.3 (53.0) | - | - |
| **dATP #1** | I911-3 | 1.0000 | a = 78 Å  b = 94 Å  c = 87 Å  β = 112 ° | 29.2 - 1.96  (2.08 – 1.96) | 82 717  (13 204) | 99.1  (98.7) | 4.3  (4.2) | 14.8 (2.1) | 99.9 (86.4) | 7.7 (63.4) | 8.8 (72.5) | - | - |
| **dATP/CTP** | I911-3 | 0.9800 | a = 78 Å  b = 95 Å  c = 87 Å  β = 112 ° | 29.3 –2.42  (2.57 –2.42) | 44 053  (6 815) | 98.8  (95.5) | 4.2  (3.9) | 12.0 (2.0) | 99.5 (73.2) | 12.0 (72.3) | 13.7 (84.0) | - | - |
| **Native**  **(citrate, PEG400)** | ID23-2 | 0.8726 | a = 79 Å  b = 98 Å  c = 87 Å  β = 112 ° | 42.6 - 2.12 (2.25 - 2.12) | 68 379  (10 870) | 99.3  (98.0) | 3.6  (3.6) | 14.8 (2.1) | 99.9 (86.5) | 5.5 (53.7) | 6.4 (63.0) | - | - |
| **dATP #2 (Mn soak)** | I911-3 | 1.8600 | a = 92 Å  b = 95 Å  c = 137 Å  β = 96 ° | 29.4 - 2.10  (2.23 - 2.10) | 268 158  (42 473) | 99.3  (97.8) | 3.6  (3.5) | 10.1 (2.11) | 99.8 (85.8) | 7.9 (52.8) | 9.3 (62.6) | - | 0.806 (0.698) |
| **dATP #3 (Zn peak)** | I911-3 | 1.2822 | a = 92 Å  b = 95 Å  c = 137 Å  β = 97 ° | 29.4 - 2.00  (2.12 - 2.00) | 309 933  (49 601) | 99.4  (98.7) | 3.7  (3.4) | 12.3 (2.0) | 99.9 (81.9) | 7.3 (56.2) | 8.6 (66.4) | - | 0.812 (0.696) |
| **dATP #3 (high energy side of Fe peak)** | I911-3 | 1.7244 | a = 93 Å  b = 95 Å  c = 137 Å  β = 96 ° | 29.4 - 2.79  (2.96 - 2.79) | 113 593  (17 900) | 98.3  (96.1) | 3.7  (3.7) | 11.8 (2.7) | 99.7 (89.0) | 8.7 (44.1) | 10.2 (51.5) | - | 0.839 (0.659) |

Friedel pairs are treated as the same reflection, except for the peak/inflection/remote/dATP #2/dATP #3 datasets where they are treated as different reflections. The space group was P2_1_ for all datasets. Values in parentheses are for the highest resolution bins.
