# Supplementary material for: The Crystal Structure of Thermotoga maritima Class III Ribonucleotide Reductase Lacks a Radical Cysteine Pre-Positioned in the Active Site
Source: PLoS One. 2015 Jul 6;10(7):e0128199. doi: 10.1371/journal.pone.0128199 (PMC4493059; doi:10.1371/journal.pone.0128199)
Supplement: S2 Table — Numbers in parentheses are for the highest resolution bins, unless otherwise noted. (DOCX) [file pone.0128199.s010.docx]

**S2 Table:** Refinement and model quality statistics for five tmNrdD structures deposited in the PDB. Numbers in parentheses are for the highest resolution bins, unless otherwise noted.

|  | **Native**  **(citrate, glycerol)** | **Native**  **(MES)** | **Native**  **(citrate, PEG400)** | **dATP #1** | **dATP/CTP** |
| --- | --- | --- | --- | --- | --- |
| **PDB code** | 4COI | 4COM | 4CON | 4COL | 4COJ |
| **ligands** | glycerol, Zn | MES, Zn, PEG | citrate | dATP, Mg, Zn | dATP, CTP, Mg, Zn |
| **resolution range (Å)** | 24.3 – 1.94  (1.98 – 1.94) | 29.4 – 1.92  (2.00 – 1.92) | 42.6 – 2.12  (2.25 – 2.12) | 29.2 – 1.96  (2.08 – 1.96) | 29.3 – 2.48  (2.53 – 2.48) |
| **R_model_ (F) [%]** | 18.2 (33.8) | 15.4 (22.9) | 18.3 (29.3) | 19.5 (29.1) | 20.4 (28.5) |
| **R_free_ (F) [%]** | 21.7 (36.7) | 18.2 (28.3) | 21.8 (34.9) | 22.3 (30.8) | 24.2 (32.0) |
| **reflections used in refinement**  **(used for R_free_)** | 87 864  (2 210) | 95 838  (2 410) | 68 347  (1 712) | 82 656  (2 078) | 41 469  (1 050) |
| **no. of non-H atoms refined** | 10496 | 10622 | 9230 | 10075 | 10096 |
| **Wilson B-factor (Å^2^)** | 27.8 | 28.0 | 36.5 | 24.9 | 31.5 |
| **Median B-factors** |  |  |  |  |  |
| **protein** | 27 | 30 | 57 | 46 | 49 |
| **ligands** | 46 (glycerol)  35 (Zn) | 61 (PEG),  35 (MES)  27 (Zn) | 68 (citrate) | 41 (dATP)  57 (Zn)  39 (Mg) | 31 (dATP)  45 (CTP)  41 (Mg)  64 (Zn) |
| **Ramachandran favoured**  **(additionally allowed) [%]** | 98.4  (1.6) | 98.6  (1.3) | 97.9  (2.1) | 98.0  (2.0) | 97.0  (2.9) |
| **rotamer outliers [%]** | 0.83 | 1.03 | 0.93 | 0.67 | 0.66 |
| **MolProbity clash score** | 1.90 | 1.01 | 1.55 | 2.06 | 1.22 |
| **bond angle rmsd from ideal (°)** | 0.995 | 1.477 | 1.093 | 1.047 | 0.786 |
| **bond length rmsd from ideal (Å)** | 0.006 | 0.020 | 0.009 | 0.006 | 0.006 |
| **water molecules** | 469 | 712 | 188 | 355 | 181 |
