# Supplementary material for: The Crystal Structure of Thermotoga maritima Class III Ribonucleotide Reductase Lacks a Radical Cysteine Pre-Positioned in the Active Site
Source: PLoS One. 2015 Jul 6;10(7):e0128199. doi: 10.1371/journal.pone.0128199 (PMC4493059; doi:10.1371/journal.pone.0128199)
Supplement: S3 Table — Unique ribonucleotide reductase catalytic subunits were selected based on DALI [53] results using 4COI (chain A) as a search structure. The structures were pairwise superimposed using the SSM algorithm [54] in SUPERPOSE from the CCP4 suite. RMSD values for equivalent Cα positions are listed in Å and in parenthesis the number of superimposed CA positions is given. 4COI_A: Thermotoga maritima class III (607 Cα), 1H7B_A: Bacteriophage T4 class III (534 Cα), 3O0O_B: Thermotoga maritima class II (626 Cα), 3HNC_A: Homo sapiens class Ia (714 Cα), 1L1L_B: Lactobacillus leichmannii class II (717 Cα), 2CVX_A: Saccharomyces cerevisiae class Ia (664 Cα), 1PEU_A: Salmonella typhimurium class Ib (692 CA) and 1RLR_A: Escherichia coli class Ia (737 Cα). (DOCX) [file pone.0128199.s011.docx]

**S3 Table:** Structural comparison of all known large subunits of ribonucleotide reductases.

|  | **4COI_A** | **1H7B_A** | **2CVX_A** | **3HNC_A** | **1L1L_B** | **3O0O_B** | **1RLR_A** | **1PEU_A** |
| --- | --- | --- | --- | --- | --- | --- | --- | --- |
| **4COI_A** | - | - | - | - | - | - | - | - |
| **1H7B_A** | 2.34 (426) | - | - | - | - | - | - | - |
| **2CVX_A** | 3.06 (344) | 3.00 (336) | - | - | - | - | - | - |
| **3HNC_A** | 3.07 (344) | 2.92 (322) | 0.88 (640) | - | - | - | - | - |
| **1L1L_B** | 3.08 (328) | 3.20 (316) | 2.12 (409) | 1.95 (406) | - | - | - | - |
| **3O0O_B** | 3.14 (345) | 3.16 (334) | 1.74 (532) | 1.68 (529) | 1.85 (416) | - | - | - |
| **1RLR_A** | 3.25 (339) | 3.04 (312) | 1.78 (565) | 1.83 (632) | 1.96 (416) | 1.85 (495) | - | - |
| **1PEU_A** | 3.33 (344) | 2.97 (313) | 2.05 (557) | 1.99 (579) | 2.20 (425) | 2.03 (485) | 1.87 (594) | - |

Unique ribonucleotide reductase catalytic subunits were selected based on DALI [[29](#_ENREF_29)] results using 4COI (chain A) as a search structure. The structures were pairwise superimposed using the SSM algorithm [[30](#_ENREF_30)] in SUPERPOSE from the CCP4 suite. RMSD values for equivalent Cα positions are listed in Å and in parenthesis the number of superimposed CA positions is given. 4COI_A: *Thermotoga maritima* class III (607 Cα), 1H7B_A: Bacteriophage T4 class III (534 Cα), 3O0O_B: *Thermotoga maritima* class II (626 Cα), 3HNC_A: *Homo sapiens* class Ia (714 Cα), 1L1L_B: *Lactobacillus leichmannii* class II (717 Cα), 2CVX_A: *Saccharomyces cerevisiae* class Ia (664 Cα), 1PEU_A: *Salmonella typhimurium* class Ib (692 CA) and 1RLR_A: *Escherichia coli* class Ia (737 Cα).
