# Supplementary material for: The Crystal Structure of Thermotoga maritima Class III Ribonucleotide Reductase Lacks a Radical Cysteine Pre-Positioned in the Active Site
Source: PLoS One. 2015 Jul 6;10(7):e0128199. doi: 10.1371/journal.pone.0128199 (PMC4493059; doi:10.1371/journal.pone.0128199)
Supplement: S1 Text — (DOCX) [file pone.0128199.s012.docx]

# Supporting Appendix: The crystal structure of *Thermotoga maritima* class III ribonucleotide reductase lacks a radical cysteine pre-positioned in the active site

Oskar Aurelius^1^, Renzo Johansson^1^, Viktoria Bågenholm^1^, Daniel Lundin^2^, Fredrik Tholander^3^, Alexander Balhuizen^1±^, Tobias Beck^4,#^, Margareta Sahlin^2^, Britt-Marie Sjöberg^2^, Etienne Mulliez^5^ & Derek T. Logan^1^

*^1^Dept of Biochemistry & Structural Biology, Lund University, Box 124, S-221 00 Lund, Sweden; ^2^Dept. of Biochemistry & Biophysics, Stockholm University, S-106 91 Stockholm, Sweden; ^3^Dept. of Medical Biochemistry and Biophysics, Karolinska Institute, Solna, Sweden; ^4^Dept. of Inorganic Chemistry, Georg-August Universität Göttingen, Germany; ^5^LCBM, Groupe de Biocatalyse, CEA-Grenoble, Institut de Recherches en Technologies et Sciences pour le Vivant (iRTSV), 38054 Grenoble Cedex 09, France*

± Current address: Laboratory of Experimental Medicine, ULB Centre for Diabetes Research, Université Libre de Bruxelles, 1070 Brussels, Belgium.
^#^ Current address: RWTH Aachen, Institute of Inorganic Chemistry, Landoltweg 1, 52074 Aachen, Germany

## Materials and Methods

***Expression and purification of tmNrdD:*** The pRSFduet-1 plasmid containing the Tm *nrdD* gene cloned into the Nco1 and HindIII restriction sites of the vector was used to transform chemically competent cells from the *E. coli* strain BL21-CodonPlus(DE3)-RIL (Agilent Technologies) using heat shock at 42°C for 30 s. These cells were streaked on to agar plates and single colonies were inoculated in 10 mL LB medium overnight at 37°C. These 10 mL were used to inoculate 1 L of LB medium containing chloramphenicol (100 μg/mL) and kanamycin (30 μg/mL). The culture was incubated in an orbital shaker (200 rpm) at 37 °C until absorbance at 600 nm reached 0.5-0.7. Protein expression was induced at 30 °C by adding IPTG to a final concentration of 250 μM, and the culture was continued for 3 hours at that temperature. The cells were harvested by centrifugation at 6000 rpm for 20 min. and washed in 50 mM Tris pH 8. The cell pellets were dissolved in 25 mL of lysis buffer (50 mM Tris•HCl, 250 mM KCl, 200 μM PMSF, 5 mM dithiothreitol (DTT), pH 8) and lysed using a French press. Cell debris was removed by centrifugation at 20 000 g for 20 minutes. The clear bacterial extract was heated to 75°C for 10 minutes and the denatured proteins removed by centrifugation (10 000 g, 10 min). Ground ammonium sulphate was then added to the supernatant to a concentration of 60% to precipitate the remaining proteins, including tmNrdD. The precipitated proteins were collected by centrifugation at 10 000 g for 10 min. The pellets were dissolved in 25 mL buffer A (50 mM Tris•HCl, 50 mM KCl, 1M ammonium sulphate, pH 8) and clarified by centrifugation. The supernatant was run on a butyl Sepharose HiTrap FF column, 5 mL (GE Healthcare), where it was eluted with a gradient from 0-100% Buffer B (50 mM Tris•HCl, 50 mM KCl, pH 8) at a flow rate of 5 mL/min. All fractions from the appropriate protein peak were pooled. The samples were concentrated in 15 mL concentration tubes with a molecular weight cutoff of 30 kDa (Millipore). As a final step the concentrated protein was run with Buffer B on a 23 mL Superdex 200 column (GE Healthcare) at a flow rate of 0.5 mL/min. TmNrdD appeared as a large peak at about 10 mL after addition of the protein, and suitable fractions were pooled and concentrated to 25 mg/mL. The protein was aliquoted into 20-30 μL portions, flash frozen in liquid nitrogen and stored at -80°C.

*Mutants C329A, C330A and double mutant C329A/C330A:* The mutants were generated using the QuikChange Lightning Kit (Agilent, France) and primers synthesized by Eurofins (Germany). The primers were:

C329A (forward): gac tcg ata gat gct gtg gcg tcc gcc tgc agg ctc aca tcg tct
C329A (reverse): aga cga tgt gag cct gca ggc gga cgc cac agc atc tat cga gtc
C330A (forward): gac tcg ata gat gct gtg gcg tcc tgc gcc agg ctc aca tcg tct
C330A (reverse): aga cga tgt gag cct ggc gca gga cgc cac agc atc tat cga gtc C329A/C330A (forward): gac tcg ata gat gct gtg gcg tcc gcc gcc agg ctc aca tcg tct C329A/C330A (reverse): aga cga tgt gag cct ggc ggc gga cgc cac agc atc tat cga gtc

***Native and Se-Met tmNrdD crystallization:*** Optimal crystallization conditions were identified in screens carried out at the Protein Crystallization Facility in Lund that were designed to test the effect of the additives B3C and I3C [[1](#_ENREF_1)] on the crystallizability of a variety of proteins. The commercial screens JCSG+ and PACT Premier (Molecular Dimensions Ltd., Newmarket, UK) were used. Well-diffracting crystals that had not been seen in previous screens were obtained both with and without B3C and I3C. Extensive optimization was carried out around the initial hits. All crystallization was done by vapour diffusion, either with sitting or hanging drops. Conditions for SeMet protein were found using a similar approach. Initially native tmNrdD was crystallized at 21 °C with a protein concentration of 24 mg/mL in 8% (w/v) PEG3000, 100 mM Na citrate buffer pH 5.3, 5 mM DTT. SeMet-tmNrdD crystallized at 24 mg/mL in 12% (w/v) PEG3000, 100 mM citrate pH 5.3, 5 mM DTT (also at 21 °C). The protein buffer was in all these cases 50 mM Tris pH 8.0 and 50 mM KCl. Drop to reservoir rations varied between 1:1 to 2:1. Crystals measuring up to 0.30 x 0.10 x 0.05 mm appeared within 1 day and grew to full size within 1 week. For cryoprotection 20% (v/v) glycerol or PEG400 was introduced by soaking.

***Data collection:*** All diffraction data were collected at 100K after flash-cooling of the crystals in liquid N_2_ or directly in the cold gas stream from a CryoJet (Oxford Cryosystems, Oxford, UK. Native tmNrdD data with citrate buffer and glycerol as cryoprotectant were collected at station I911-2 of the MAX II synchrotron, Lund, Sweden, on a 165mm marCCD detector. Multiple wavelength anomalous diffraction (MAD) data collection for the SeMet-tmNrdD crystal was performed with an ADSC Q315R detector at station ID23-1 of the ESRF, Grenoble, France [[2](#_ENREF_2)]. An absorption edge scan of the selenium K edge was carried out. The peak wavelength was collected first, followed by the inflection and remote wavelengths. For all datasets an oscillation range of 1° was used and 360 images collected with 0.3 s exposure time at 4% beam transmission. Helical scanning was used to lessen the effect of radiation damage. One translation along the length of the crystal was carried out for each wavelength. iMOSFLM [[3](#_ENREF_3)] was used to decide on a data collection strategy. Indexing and integration of the data were done with XDS and scaling with XSCALE [[4](#_ENREF_4)].

***Structure solution:*** POINTLESS [[5](#_ENREF_5)] was used to distinguish between space groups P2 and P2_1_. Matthews_coef [[6](#_ENREF_6)] was used for calculation of the Matthews coefficient, solvent content and likeliest composition of the asymmetric unit (two monomers considered corresponding to a Matthew’s coefficient of 2.4 Å^3^/Dalton and a solvent content of 47.6%).[[7](#_ENREF_7)]

The structure of SeMet-tmNrdD was solved to 2.5 Å resolution by MAD using the AutoRickshaw pipeline in expert mode [[8](#_ENREF_8)]. The individual data sets for each wavelength were combined and scaled by CAD and SCALEIT [[9](#_ENREF_9)]. The resolution range 20.0–2.5 Å was used for the sub-structure determination and phasing. SHELXD [[10](#_ENREF_10)] was used for substructure determination. Of 40 possible Se sites in the asymmetric unit, 23 were found and refined by MLPHARE [[11](#_ENREF_11)]. Phasing with MLPHARE gave a figure of merit of 0.493. Density modification and phase extension was performed with DM [[12](#_ENREF_12)] with a final figure of merit of 0.714. Further density modification and phase extension was performed by RESOLVE [[13](#_ENREF_13)].

***Model building and refinement:*** Automatic model building was performed within the Auto-Rickshaw pipeline. HELICAP [[14](#_ENREF_14)] modeled 622 residues which were used as a starting model for BUCCANEER [[15](#_ENREF_15)] which built 1 089 of 1 302 residues (84 %) in SeMet-tmNrdD. After refinement in CNS [[16](#_ENREF_16)] and Refmac5 [[17](#_ENREF_17)] R_model_ was 21.4% and R_free_ was 26.4%. Rigid body refinement of this model was carried out against the native (citrate and glycerol) dataset to 3 Å with Refmac5. Restrained refinement was then carried out using the full resolution range up to 1.94 Å. Manual model building was performed in Coot [[18](#_ENREF_18)], increasing the number of residues built to 1218 (94%). TLS refinement [[19](#_ENREF_19)], dividing each monomer into two TLS groups (Zn-domain residues 590-616 as one group and the remaining residues as the second group), was carried out with riding hydrogen atoms. Modelling of water molecules was done manually and with the find_waters function in Coot [[18](#_ENREF_18)]. When most electron density ≥ 1σ had been accounted for, geometry optimization was carried out using the validation functions in Coot and continuous evaluation of each refinement cycle in MolProbity [[20](#_ENREF_20)]. The X-ray weight was adjusted manually in Refmac5 to minimize the gap between R_model_ and R_free_ [[21](#_ENREF_21)]. Final refinement work as performed in Phenix [[22](#_ENREF_22)] with ADP and stereochemistry weight optimization, secondary structure restraints and automatic TLS group definitions.

Other datasets were generally solved by rigid body refinement to 4 Å in Refmac5 using the native (citrate and glycerol) model followed by restrained refinement to full resolution. Final refinement steps were performed in phenix.refine. Reflections for R-free were imported from the native (citrate and glycerol) data before refinement.

***Nucleotide complexes:*** The dATP/CTP complex was obtained by soaking a crystal of native tmNrdD (20 mg/mL tmNrdD, 100 mM MES pH 6.1, 16% [w/v] PEG3000 and 5 mM DTT) in 0.5 mM dATP, 2 mM CTP, 10 mM MgCl_2_ and 20% (v/v) PEG400 for approximately 3 hours prior to flash-cooling. A similar soaking was prepared for the dATP dataset, but excluding the CTP. The structures were solved and refined as described in the general format above.

***Metal characterization:*** To verify the modelling of magnesium coordination in the effector site, an equivalent dATP soak as above was prepared, but with the Mg^2+^ replaced by Mn^2+^. For data collection the X-ray wavelength was chosen to be on the high-energy side of the Mn^2+^ K-edge.

To determine whether the C-terminal metal site contained Fe or Zn, a dATP and Mg^2+^ soaked crystal (as discussed in the *Nucleotide complexes* section) was used to collect two datasets at the high-energy side of the Zn or Fe K-edges respectively. The anomalous difference maps only showed strong peaks at the Zn K-edge, but not by the Fe K-edge, indicating that the C-terminal metal site coordinates a Zn^2+^ ion.

The metal characterization data sets were processed with Friedel pairs treated as unique reflections. The structures were solved with Phaser [[23](#_ENREF_23)] due to different crystal packing with 4 monomers per asymmetric unit instead of 2. Further processing and model treatment was otherwise the same as in the general case, but also included the preparation of anomalous difference map.

***SDS-PAGE analysis of dissolved crystals:*** More than 30 small crystals were removed from crystallization drops after two days and were washed sequentially in three drops containing 15 μl reservoir solution before dissolving in 10 μl of 0.1 M MES buffer pH 7.5, 5 mM DTT. Loading buffer for SDS PAGE was added. As controls, protein taken directly from storage at -80 °C was analyzed, as well as protein that had been suspended in a drop without added precipitant over its own buffer as reservoir, in order to “age” it for the same time as the drop that produced crystals. The gel was run, stained and destained using standard protocols.

***Construct design and cloning for soluble MBP-tmNrdG:*** A synthetic tmNrdG gene was ordered from GenScript (Piscataway, NJ). Codon optimization for *E. coli* and cloning into pUC57 was performed by GenScript. Further cloning, protein expression and purification was performed by Lund Protein Production Platform (LP3). A mutation corresponding to R143Q was introduced based on the observation that the database translated sequence (UniProt entry Q9WYL5) deviated from sequencing results of PCR products from *Thermotoga maritima* genomic DNA and the fact that this residue is consistently a glutamine in all other *Thermotogales*. The tmNrdG gene was cloned into the expression vector pETM_44-ccdB via sequence- and ligase independent cloning. The resulting plasmid, pET44_nrdG, was verified by DNA sequencing (Eurofins MWG Operon). pET44_nrdG was transformed into electrocompetent *E. coli* TUNER (DE3) cells together with the plasmid pDB1282, a kind gift of Prof. Dennis Dean. The plasmid pDB1282 contains the *iscS-iscU-iscA-hscB-hscA-fdx* portion of the *isc* operon from *Azotobacter vinelandii* under the control of an arabinose inducible promoter. The *iscS* gene encodes a cysteine desulphurase, which activates the sulphur of L-cysteine for use in the formation of FeS clusters. The iscU and iscA genes are believed to encode proteins that serve as scaffolds for the construction of precursors of mature FeS clusters, while the hscB and hscA genes encode molecular chaperones that facilitate incorporation of the FeS clusters into the apoprotein. The *fdx* gene encodes a ferrodoxin that helps maintaining electron balance during formation of FeS clusters [[24](#_ENREF_24)]. pDB1282 also confers ampicillin and kanamycin resistance.

The construct prepared in pET44_nrdG introduced *E. coli* maltose binding protein (UniProt entry K0BGG6) with a 6x His-tag as an N-terminal fusion to tmNrdG in order to increase solubility of tmNrdG.

***Expression and purification of MBP-tmNrdG:*** All expression and purification procedures were performed aerobically. 1 L of Terrific Broth supplemented with 100 μM FeCl_3_, 100 mg/L ampicillin and 50 mg/L kanamycin was inoculated to OD 600 = 0.1 with an overnight culture of *E. coli* TUNER (DE3) pDB1282, pET44_nrdG. The culture was grown in a 5 L Erlenmeyer flasks with indentations at 30°C, 120 rpm shaking. At OD_600_ = 0.3, arabinose was added to a final concentration of 0.05 % (w/v) to induce expression from the pDB1282 plasmid. At OD_600_ = 0.7, IPTG was added to a final concentration of 0.4 mM to induce MBP-tmNrdG production. 4 h 45 min after IPTG induction, cells were harvested in a JA10 rotor (8 000 g, 15 min, 4 °C, 500 mL/centrifuge flask) and the pellets were stored at -80 °C. The cell pellet was resuspended in 25 mL 50 mM NaPO_4_, 300 mM NaCl, 20 mM imidazole, pH 8.0, supplemented with 1 tablet Complete protease inhibitor, EDTA free (Roche) and passed twice through a French Pressure Cell at 18 000 psi. The resulting lysate was ultracentrifuged in a Ti 50.2 rotor, 45 000 rpm, 60 min, 4°C. The supernatant was passed through a 0.45 µm filter and used for affinity chromatography. A 1 mL HisTrap HP column (GE Healthcare) was connected to an ÄKTA Avant system (GE Healthcare). The flow rate was set to 1 mL/min. The column was washed with 5 column volumes dH_2_O and was then equilibrated with 10 column volumes 50 mM NaPO_4_, 300 mM NaCl, 20 mM imidazole, pH 8.0. The sample was applied and the column was washed with 50 mM NaPO_4_, 300 mM NaCl, 20 mM imidazole, pH 8.0 until a stable UV signal was obtained. The bound protein was eluted with a linear 0-100% gradient of 50 mM NaPO_4_, 300 mM NaCl, 500 mM imidazole, pH 8.0 over 20 column volumes. During elution 1 mL fractions were collected. The chromatography run was performed at room temperature while the fractions were collected at 6 °C.

Peak fractions were pooled and dialyzed in a Spectra/Por dialysis membrane, MWCO 6-8000, width 32 mm, against 1 L 50 mM Tris, 150 mM KCl, pH 8.0 at 4 °C overnight. In the morning, the dialysis tubing was transferred to a fresh 1 L 50 mM Tris, 150 mM KCl, pH 8.0 and the dialysis was continued for 2 h.

The dialyzed protein was concentrated using an Amicon ultracentrifugal filter (MW CO 30 kDa) and the protein concentration was determined using a Nanodrop spectrophotometer. The purity was estimated to be > 80% based on SDS-PAGE analysis.

***Reconstitution of the 4Fe-4S cluster in MBP-tmNrdG:*** All reconstitution steps and further work with *holo* MBP-tmNrdG were performed anaerobically in a glove box (Jacomex NT) in a N_2_ atmosphere at 18–24°C. Reconstitution batches were of varying volume (0.5 – 1.5 mL) with 100 μM MBP-tmNrdG in 100 mM Tris pH 8, 100 mM KCl and 2 mM DTT. An eightfold molar excess of L-cysteine and a sixfold molar excess of ammonium Fe(II) sulphate were added. The reconstitution was started by the addition of 2 μM cysteine desulphurase and followed spectroscopically by 400 nm absorbance. Reconstitution times were normally around two hours, after which the sample was desalted on a NAP-25 column (GE Healthcare) and concentrated with Amicon® ultracentrifugal filters (Millipore). The reconstituted MBP-tmNrdG was frozen in liquid nitrogen and stored at -80°C.

***EPR sample preparation:*** For EPR verification of tmNrdD activation, a 1:1 molar incubation (120 µM per protein) of tmNrdD and *holo* MBP-tmNrdG was prepared. This incubation included 18 µM 5-deazaflavin and 5 mM DTT in 100 µl of 100 mM Tris-HCl pH 8.0, 100 mM KCl in H_2_O under anaerobic conditions. The incubation was illuminated for 15 minutes with a slide projector, after which 1.3 mM SAM was added and incubated for 10 minutes before freezing in isopentane cooled by liquid nitrogen. X-band EPR spectra were recorded on a Bruker ESP-4116 dual mode cavity and an Oxford Instruments ESR-9 flow cryostat. Measurements were performed at 20 K under non-saturating conditions (2 µW microwave power at 9.65 GHz frequency).

A second 1:1 incubation (120 µM per protein) of tmNrdD and *holo* MBP-tmNrdG was prepared in 100 µl 100 mM Tris-HCl pH 7.5, 100 mM NaCl in D_2_O**.** Protein stock solutions were in H_2_O. The incubation was diluted with the D_2_O containing buffer and concentrated with Amicon® Ultra centrifugal filters (Millipore) in multiple cycles, corresponding to a final H_2_O buffer content of < 1%. The volume was adjusted to 100 µl again with D_2_O buffer, but no adjustments were made for protein losses in the centrifugation filters. 5 mM DTT and 18 µM 5-deazaflavin (H_2_O stock solutions, < 2% of final volume) were added and the incubation was illuminated for 15 minutes with a slide projector. After the illumination 1.3 mM SAM was added (H_2_O stock solution, < 3% of final volume) and incubated for 15 minutes before freezing in isopentane cooled by liquid nitrogen. Measurements were performed as above.

***Enzymatic activity assays:*** Assays in a volume of 100 µL were run in two consecutive steps. Firstly tmNrdD (4 µM) was activated in a glove box at 37°C in the presence of *holo* MBP-tmNrdG (2.5 µM) in a Tris-KCl buffer pH 8.0 containing anaerobically prepared *T. maritima* cell extracts (200 µg), DTT (5 mM), S-adenosylmethionine (150 µM) and deazaflavin (5 µM) under white light illumination for 30 min. In a second step, ^3^H-CTP substrate (1 mM, 17 000 dpm*nmol^-1^) was added to the activated protein along with dATP (2.5 mM) and Mg^2+^ (8 mM) and illumination was maintained during the time of the reaction (20-60 min). The reaction mixture was taken out of the box and mixed with 1 M perchloric acid and dCMP as internal standard. The solution was heated to 100°C for 15 min to generate the nucleoside monophosphate. After neutralization with KOH and centrifugation the supernatant was loaded onto a 6 mL Dowex-50X8 column (H^+^ form). The unreacted CMP was eluted with 106 mL acetic acid (0.2 M) and dCMP was recovered in the following 30 mL. The amount of dCMP, and hence of dCTP, was determined by scintillation counting after correction for the yield of the Dowex column.

Activity assays of the mutants followed the same general protocol as for the wild type. In experiment 1, assays in a volume of 100 µl were run in two consecutive steps with the following parameters: tmNrdD (20 µM) was activated in a glove box at 37°C in the presence of *holo*-MBP-tmNrdG (2.5 µM) in a Tris-KCl buffer pH 8.0 containing anaerobically prepared *T. maritima* cell extracts (140 µg), DTT (5 mM), S-adenosylmethionine (150 µM) and deazaflavin (5 µM) under white light illumination for 30 min. In a second step, ^3^H-CTP substrate (1 mM, 17 000 dpm*nmol^-1^) was added to the activated protein along with dATP (2 mM) and Mg^2+^ (8 mM) and illumination was maintained during the time of the reaction (20 min). Quenching of the reaction and further treatment of samples as described for previous assays.

In experiment 2 assays were performed with ^3^H-CTP or cold CTP. The ^3^H-CTP was performed once in parallel with the cold CTP series. On a separate occasion the cold CTP assay was repeated with two samples per condition. Assays in a volume of 80 µl were run in two consecutive steps with the following parameters: tmNrdD (5 µM) was activated in a glove box at 27°C in the presence of *holo*-MBP-tmNrdG (5 µM) in a Tris-KCl buffer pH 7.6 containing anaerobically prepared *T. maritima* cell extracts (140 µg), DTT (5 mM), S-adenosylmethionine (1 mM) and deazaflavin (8 µM) under white light illumination for 30 min. In a second step, ^3^H-CTP substrate (1 mM, 49 600 cpm*nmol^-1^) or cold CTP (1 mM) was added to the activated protein along with dATP (4 mM) and Mg^2+^ (10 mM) and illumination was maintained during the time of the reaction (40 min). The reaction mixtures were taken out of the box and exposed to oxygen. The ^3^H-CTP samples were mixed with 1 M perchloric acid and dCMP as internal standard. Both the ^3^H-CTP and cold CTP assay series were heated to 100°C for 10 min. The ^3^H-CTP assay series was neutralized with KOH. Both assay series were centrifuged and the supernatant was used for chromatography. Further treatment of the ^3^H-CTP assay samples followed the same protocol as previously described.

The enzymatic activity of samples from the assay series with cold CTP was determined using HPLC-based analysis. The supernatant from centrifugation was diluted in water (1:1) and injected into an HPLC system (Agilent 1260 Infinity Bio-inert Quaternary LC system) connected to a Waters Symmetry C_18_ column (4.6 x 150 mm, 3.5 µm, 100 Å). The column was eluted at 0.5 mL/min with a 30 min gradient of increasing (10-20%) methanol in 50mM phosphate buffer (pH 7) supplemented with tetrabutylammonium hydroxide (10mM), which was then held constant at 20 % methanol for another 15min. Eluted material was monitored by measuring the UV absorbance at 270nm and by collection of absorption spectra between 190-400 nm throughout the whole run. Peak identity was verified by comparison with the retention times and spectra of injected standards. Quantification was based on peak height measurements with the peak for dATP (the constant part of the reaction mixture for all samples) as an internal reference. An average of the results from the three measurements, per unique condition, for the cold CTP assay was used.

The detection limit of the ^3^H-CTP assay is estimated to be two times the blank sample, which would correspond to 35 pmol. Determination of detection limit (10 pmol) and linear range (10–500 pmol) for the HPLC assay was made through the injection of standards.

The cold CTP assay was also used to test for activity in the presence of the *E. coli* thioredoxin system. The thioredoxin was a kind gift from Prof. Arne Holmgren, Karolinska Institute, Stockholm, Sweden. Equivalent assay reactions were prepared lacking *T. maritima* cell extracts and instead containing 1 mM NADPH, 1 µM thioredoxin reductase and 30 µM thioredoxin. These assay reactions showed no detectable amount of dCTP.

***Phylogenetics and classification:*** In version 2 of the RNRdb database (in beta at [http://rnrdb.pfitmap.org](http://rnrdb.pfitmap.org/)) a new classification scheme for RNRs is proposed based on phylogenetic analyses of available structures and sequences. We downloaded all 70 NrdDh sequences from whole genome sequenced organisms, including two phages, present in the NCBI RefSeq database (October 2013), and aligned them using ProbCons [[25](#_ENREF_25)]. One sequence (NCBI accession number ZP_06092034) was deleted from the set due to questionable sequence quality. The two phages had inteins in their sequences (inserted in the SCCR motif) that were removed prior to alignment.

To investigate the sequence space within the NrdDh subclass, the alignment was analyzed with the NeighborNet [[26](#_ENREF_26)] and BioNJ [[27](#_ENREF_27)] algorithms in SplitsTree4 [[28](#_ENREF_28)]. The phylogenetic network in S6 Fig. allowed us to propose four distinct groups of NrdDh proteins: NrdDh1-4, delineated by the splits marked with dashed red lines. The two phage sequences are outside the diversity described by the groups, but were deemed too few and similar to represent their own group. The four groups were added to RNRdb2. The sequence motifs mentioned in the main text are conserved within groups as indicated in the figure.

**References**

1. Beck T, Krasauskas A, Gruene T, Sheldrick GM. A magic triangle for experimental phasing of macromolecules. Acta Crystallogr D Biol Crystallogr. 2008;64(Pt 11):1179-82. doi: 10.1107/S0907444908030266. PubMed PMID: 19020357.

2. Nurizzo D, Mairs T, Guijarro M, Rey V, Meyer J, Fajardo P, et al. The ID23-1 structural biology beamline at the ESRF. J Synchrotron Radiat. 2006;13(Pt 3):227-38. doi: 10.1107/S0909049506004341. PubMed PMID: 16645249.

3. Battye TGG, Kontogiannis L, Johnson O, Powell HR, Leslie AGW. iMOSFLM: a new graphical interface for diffraction-image processing with MOSFLM. Acta Crystallogr D Biol Crystallogr. 2011;67:271-81. doi: 10.1107/s0907444910048675. PubMed PMID: ISI:000288532800006.

4. Kabsch W. XDS. Acta Crystallogr D Biol Crystallogr. 2010;66:125-32. PubMed PMID: ISI:000273820800003.

5. Evans PR. Scaling and assessment of data quality. Acta Crystallogr D Biol Crystallogr. 2006;62(1):72-82. doi: doi:10.1107/S0907444905036693.

6. Kantardjieff KA, Rupp B. Matthews coefficient probabilities: Improved estimates for unit cell contents of proteins, DNA, and protein-nucleic acid complex crystals. Protein Science. 2003;12(9):1865-71. doi: 10.1110/ps.0350503. PubMed PMID: ISI:000184976100006.

7. Vagin A, Teplyakov A. MOLREP: an automated program for molecular replacement. J Appl Crystallogr. 1997;30:1022-5.

8. Panjikar S, Parthasarathy V, Lamzin VS, Weiss MS, Tucker PA. Auto-Rickshaw: an automated crystal structure determination platform as an efficient tool for the validation of an X-ray diffraction experiment. Acta Crystallogr D Biol Crystallogr. 2005;61:449-57. doi: 10.1107/s0907444905001307. PubMed PMID: ISI:000227867600012.

9. Howell PL, Smith GD. Identification of Heavy-Atom Derivatives by Normal Probability Methods. J Appl Crystallogr. 1992;25:81-6. doi: Doi 10.1107/S0021889891010385. PubMed PMID: WOS:A1992HD73300014.

10. Schneider TR, Sheldrick GM. Substructure solution with SHELXD. Acta Crystallogr D Biol Crystallogr. 2002;58:1772-9. doi: Doi 10.1107/S0907444902011678. PubMed PMID: WOS:000178248100002.

11. Collaborative Computational Project no. 4. The CCP4 suite: programs for protein crystallography. Acta Crystallogr D Biol Crystallogr. 1994;50(Pt 5):760-3. PubMed PMID: 15299374.

12. Cowtan KD, Zhang KY. Density modification for macromolecular phase improvement. Prog Biophys Mol Biol. 1999;72(3):245-70. Epub 1999/12/03. doi: S0079-6107(99)00008-5 [pii]. PubMed PMID: 10581970.

13. Terwilliger TC. Maximum-likelihood density modification using pattern recognition of structural motifs. Acta Crystallogr D Biol Crystallogr. 2001;57(Pt 12):1755-62. PubMed PMID: 11717487.

14. Morris RJ, Zwart PH, Cohen S, Fernandez FJ, Kakaris M, Kirillova O, et al. Breaking good resolutions with ARP/wARP. J Synchrotron Radiat. 2004;11:56-9. doi: Doi 10.1107/S090904950302394x. PubMed PMID: WOS:000188920800016.

15. Cowtan K. The Buccaneer software for automated model building. 1. Tracing protein chains. Acta Crystallogr D Biol Crystallogr. 2006;62(Pt 9):1002-11. Epub 2006/08/25. doi: S0907444906022116 [pii]

10.1107/S0907444906022116. PubMed PMID: 16929101.

16. Brunger AT. Version 1.2 of the Crystallography and NMR system. Nature Protocols. 2007;2(11):2728-33. doi: Doi 10.1038/Nprot.2007.406. PubMed PMID: WOS:000253140000009.

17. Murshudov GN, Vagin AA, Dodson EE. Refinement of macromolecular structures by the maximum-likelihood method. Acta Crystallogr D Biol Crystallogr. 1997;53:240–55.

18. Emsley P, Lohkamp B, Scott WG, Cowtan K. Features and development of Coot. Acta Crystallogr D Biol Crystallogr. 2010;66(Pt 4):486-501. Epub 2010/04/13. doi: S0907444910007493 [pii]

10.1107/S0907444910007493. PubMed PMID: 20383002; PubMed Central PMCID: PMC2852313.

19. Winn MD, Murshudov GN, Papiz MZ. Macromolecular TLS refinement in REFMAC at moderate resolutions. Methods Enzymol. 2003;374:300-21. PubMed PMID: 14696379.

20. Chen VB, Arendall WB, 3rd, Headd JJ, Keedy DA, Immormino RM, Kapral GJ, et al. MolProbity: all-atom structure validation for macromolecular crystallography. Acta Crystallogr D Biol Crystallogr. 2010;66(Pt 1):12-21. Epub 2010/01/09. doi: 10.1107/S0907444909042073. PubMed PMID: 20057044; PubMed Central PMCID: PMC2803126.

21. Laskowski RA, MacArthur MW, Moss DS, Thornton JM. PROCHECK: A program to check the stereochemical quality of protein structures. J Appl Crystallogr. 1993;26:283-91.

22. Adams PD, Afonine PV, Bunkoczi G, Chen VB, Davis IW, Echols N, et al. PHENIX: a comprehensive Python-based system for macromolecular structure solution. Acta Crystallogr D Biol Crystallogr. 2010;66(Pt 2):213-21. Epub 2010/02/04. doi: DOI 10.1107/S0907444909052925. PubMed PMID: 20124702; PubMed Central PMCID: PMC2815670.

23. McCoy AJ, Grosse-Kunstleve RW, Adams PD, Winn MD, Storoni LC, Read RJ. Phaser crystallographic software. J Appl Crystallogr. 2007;40(Pt 4):658-74. Epub 2007/08/01. doi: 10.1107/S0021889807021206. PubMed PMID: 19461840; PubMed Central PMCID: PMC2483472.

24. Zheng L, Cash VL, Flint DH, Dean DR. Assembly of iron-sulfur clusters. Identification of an iscSUA-hscBA-fdx gene cluster from *Azotobacter vinelandii*. J Biol Chem. 1998;273(21):13264-72. Epub 1998/05/28. PubMed PMID: 9582371.

25. Do CB, Mahabhashyam MS, Brudno M, Batzoglou S. ProbCons: Probabilistic consistency-based multiple sequence alignment. Genome Res. 2005;15(2):330-40. Epub 2005/02/03. doi: 15/2/330 [pii] 10.1101/gr.2821705. PubMed PMID: 15687296; PubMed Central PMCID: PMC546535.

26. Bryant D, Moulton V. Neighbor-net: an agglomerative method for the construction of phylogenetic networks. Mol Biol Evol. 2004;21(2):255-65. doi: 10.1093/molbev/msh018. PubMed PMID: 14660700.

27. Gascuel O. BIONJ: an improved version of the NJ algorithm based on a simple model of sequence data. Mol Biol Evol. 1997;14(7):685-95. PubMed PMID: 9254330.

28. Huson DH, Bryant D. Application of phylogenetic networks in evolutionary studies. Mol Biol Evol. 2006;23(2):254-67. doi: 10.1093/molbev/msj030. PubMed PMID: 16221896.
